# Hormone-Mediated Signaling of Reactive Oxygen Species Under Abiotic Stress

**DOI:** 10.3390/ijms27219656

**Published:** 2026-10-28

**Authors:** Larisa I. Fedoreyeva, Sergey A. Bursakov, Tatiana P. Molchanova, Oksana A. Muratova, Ekaterina V. Zakharova

**Affiliations:** All-Russia Research Institute of Agricultural Biotechnology, Moscow 127550, Russia; sergeymoscu@gmail.com (S.A.B.); tatihan@gmail.com (T.P.M.); muratova.biology@gmail.com (O.A.M.); zakharova_ekater@mail.ru (E.V.Z.)

**Keywords:** ROS, ROS signaling, Ca^2+^ signaling, phytohormones, abiotic stress

## Abstract

Abiotic stresses trigger the generation of reactive oxygen species (ROS) in various subcellular compartments of plant cells, where they perform a dual function: acting as cytotoxic agents during acute stress and as highly specific signaling modulators under mild or chronic stress. However, the fundamental question of how the cell distinguishes between toxic and signaling ROS pools—and how phytohormones coordinate this distinction—remains unresolved. This review re-examines the molecular logic of the hormone-redox interface, focusing on the post-translational modifications of NADPH oxidases (RBOHs) and antioxidant enzymes as a key mechanism for the rapid tuning of signals. Moving beyond traditional linear cascade models, we propose the concept of a “dynamic hormonal code,” in which abscisic acid acts as the primary initiator of apoplastic ROS bursts via SnRK2-dependent phosphorylation, while salicylates and jasmonates modulate the duration and amplitude of these bursts, thereby determining the outcome—ranging from programmed cell death to systemic resistance. Known mechanisms of the hormonal code have been established (e.g., ABA → SnRK2 → RBOH; SA/JA antagonism mediated by NPR1; and the redox relay involving PRXIIB/GPX3 → ABI2). This review goes beyond simple hormone combinatorics by proposing a conceptual framework: the hormonal code encodes ROS signals through parameters of time, amplitude, and cellular compartment. Particular attention is paid to recent discoveries regarding the roles of auxin and brassinosteroids in reconfiguring chloroplast redox homeostasis during flooding and heat shock. Generalizing these data, we propose an integrated model of spatiotemporal regulation that explains how feedback loops involving ROS, Ca^2+^ waves, and hormone receptors facilitate systemic acclimation. Thus, targeting key crosstalk nodes—specifically SnRK2 and TOR kinase complexes—offers new strategies for engineering stress-resilient crops in the face of climate change.

## 1. Introduction: Integration of Redox and Hormonal Networks as a Key to Plant Stress Tolerance

Under natural conditions, plants are constantly exposed to adverse environmental factors that cause abiotic stresses, which pose a serious threat to agricultural productivity [1,2]. The most common stressors include drought, soil salinity, extreme temperatures (cold and heat), and flooding. These effects disrupt physiological, biochemical, and molecular processes, leading to growth inhibition, reduced photosynthetic activity, damage to cellular structures, and ultimately, significant yield losses [3,4,5,6].

Drought causes tissue dehydration, disruption of water and osmotic balance, decreased turgor and stomatal conductance, and oxidative damage. Soil salinization, exacerbated by irrigation and the use of chemical fertilizers, leads to ionic imbalance, osmotic stress, and toxicity [7]. Extreme temperatures disrupt membrane stability, cause protein denaturation, and metabolic imbalance [1,3,8]. Flooding creates hypoxic conditions, limiting respiration and energy metabolism [9]. Understanding the molecular mechanisms of stress responses and developing tools to improve plant resistance are priority tasks in modern plant biology.

Reactive oxygen species (ROS), which are generated as a result of redox reactions, play a central role in the response to abiotic stress [10]. Under normal conditions, cells maintain redox homeostasis—a balance between ROS production and their removal by the antioxidant system [11]. Basal ROS levels are essential for maintaining redox signaling, which regulates physiological processes of growth and development [12]. The term “redox biology” reflects the modern understanding of ROS as signaling molecules and not only as toxic agents [12]. However, under stress, ROS production increases sharply, leading to redox imbalance and oxidative stress [13]. Excess ROS can damage lipids, proteins, and DNA, but simultaneously serve as triggers for adaptive signaling cascades. Thus, fine-tuning between ROS generation and detoxification, as well as interactions with hormonal signaling pathways, determines plant survival under adverse conditions [13].

In this review, we present an integrative concept that considers ROS as central signaling molecules linking compartmentalized metabolic processes, hormonal regulation, and systemic signaling. We analyze the mechanisms of ROS generation in various subcellular compartments, the role of NADPH oxidases of the RBOH family as key generators of apoplastic ROS, the phenomenon of the ROS wave as a rapid systemic signal, and the integration of ROS signaling with Ca^2+^ signaling, electrical signals, and hormonal networks. Particular attention is paid to the role of this integrated signaling network in systemic acquired adaptation (SAA), a mechanism that allows plants to prepare uninjured tissues for upcoming stressful events.

Existing reviews describe specific branches of ROS–hormone crosstalk in detail (e.g., ABA–ROS during drought, SA–ROS during biotic stress, and auxin–ROS during root development). However, these reviews typically present linear, cascade-like models (“hormone → RBOH → ROS → response”), leaving unanswered the question of how a cell—mediated by hormones—distinguishes between toxic and signaling pools of ROS. This review offers a novel perspective based on three key points. First, we propose an explicit regulatory model—a “dynamic hormonal code”—in which ABA establishes the temporal window for a ROS signal, while SA and JA modulate its amplitude and duration through antagonism at the level of NPR1. Second, we demonstrate that signaling specificity is determined not only by the hormone involved but also by the cellular compartment (such as the apoplast, chloroplast, or peroxisome) and by redox-based modifications of key hub proteins (e.g., PRXIIB, GPX3, and HPCA1). Third, we formulate testable predictions and discuss the experimental approaches—such as genetically encoded redox biosensors, single-cell redox proteomics, and time-resolved phosphoproteomics of RBOH—that could be used to validate or refute them. Thus, rather than merely summarizing existing data, this review provides an integrative framework for their interpretation.

## 2. Generation of Reactive Oxygen Species and Compartmental Regulation

### 2.1. The Dual Nature of ROS in Plant Cells

ROS are constantly generated during plant life, particularly in photosynthetic cells. For a long time, they were considered solely as toxic metabolites causing oxidative stress. However, the modern paradigm views ROS as key components of intercellular and intracellular signaling, participating in defense mechanisms against biotic and abiotic stresses, as well as in the regulation of growth and programmed cell death.

The main forms of ROS include superoxide radicals (O_2_·^−^), hydrogen peroxide (H_2_O_2_), hydroxyl radicals (OH•), and singlet oxygen (^1^O_2_), formed through the sequential reduction of molecular oxygen [14]. Each of these forms has unique reactivity, lifetime, and signaling properties. For example, ^1^O_2_ and OH• are extremely reactive and short-lived, limiting their action to the site of generation, whereas H_2_O_2_ is more stable and can diffuse across membranes (partially via aquaporins), acting as a systemic signal.

During evolution, plants have developed a complex network of ROS generation and scavenging, controlled by a large group of genes [15]. This network functions in all cellular compartments, including chloroplasts, mitochondria, peroxisomes, the plasma membrane, the apoplast, the cell wall, and the endoplasmic reticulum. Each compartment is characterized by its own steady-state level of ROS, forming its unique “redox signature,” which varies depending on the cell type, developmental stage, and the intensity of stress. The main centers of ROS generation in plant cells are chloroplasts, peroxisomes, and mitochondria.

### 2.2. ROS Regulation in Chloroplasts: A Photosynthetic Paradox

Chloroplasts are the main sources of ROS in plant cells exposed to light. Thanks to the presence of the photosynthetic electron transport chain (ETC), these organelles are equipped with all the mechanisms for converting light energy into chemical energy, but it is precisely this activity that makes them vulnerable to electron leakage.

ROS generation in chloroplasts occurs primarily through photosystem I (PSI) and photosystem II (PSII) (Figure 1A) [16,17]. Excessive light absorption in PSII leads to the formation of the triplet state of chlorophyll (3Chl). This state inhibits photosynthesis and promotes the transfer of energy to molecular oxygen, forming highly reactive 1O2 [18,19]. Thus, PSII is the main producer of 1O2, especially under light stress. In addition to 1O2, incomplete oxidation of water in PSII can produce H2O2, which is further reduced to OH• in the presence of metal ions (Fenton reaction) [20,21,22,23].

In contrast, PSI is the primary site of O_2_ reduction to O_2_·^−^ (Mehler reaction). Under normal conditions, electrons from excited PSI reduce NADP^+^ to NADPH, which is subsequently used in the Calvin cycle for CO_2_ fixation. If the NADP^+^ pool is depleted (e.g., during stomatal closure and CO_2_ deficiency under drought conditions), direct electron transfer from ferredoxin to molecular oxygen occurs, forming O_2_·^−^ [24,25]. This process is a key photoprotective mechanism, removing excess reducing equivalents but simultaneously leading to the accumulation of ROS.

It is important to specify that PSI contributes to the chloroplastic H_2_O_2_ pool indirectly, rather than through direct two-electron reduction of O_2_. Under conditions of CO_2_ limitation—such as drought-induced stomatal closure, salinity, or high light—the acceptor side of PSI becomes over-reduced, and electrons are transferred from ferredoxin to molecular oxygen, generating O_2_·^−^ (the Mehler reaction). O_2_·^−^ is then rapidly dismutated to H_2_O_2_ by thylakoid-bound and stromal superoxide dismutase (SOD). Subsequently, H_2_O_2_ is reduced to water by thylakoid-bound and stromal ascorbate peroxidase (tAPX/sAPX), using ascorbate as the electron donor; ascorbate is regenerated through the MDHAR–DHAR–GR/GSH system, completing the water–water cycle. Because this cycle consumes electrons derived from water and returns them to water without net NADPH or ATP production, it functions as an electron sink that prevents over-reduction of the PSI acceptor side and the plastoquinone pool, thereby limiting electron leakage to O_2_ and protecting the photosynthetic apparatus from photoinhibition. At the same time, the capacity of the water–water cycle is finite: when H_2_O_2_ production exceeds APX/ascorbate regeneration capacity, H_2_O_2_ can accumulate in the stroma, modify redox-sensitive thiol enzymes, and act either as a retrograde signal or, under severe stress, as a source of oxidative damage [26]. This dual capacity is particularly relevant under drought, when ABA-induced stomatal closure lowers CO_2_ availability and simultaneously stimulates the Mehler reaction and photorespiration.

Superoxide ions (O_2_·^−^) formed in PSI are rapidly dismutated to H_2_O_2_ by superoxide dismutase (SOD), located on the thylakoid membrane. Further detoxification of H_2_O_2_ occurs through the ascorbate-glutathione cycle (water-water cycle) involving ascorbate peroxidase (APX) [27]. This cycle is critical for maintaining redox balance in the ETC and maximizing the efficiency of photosynthesis [27]. A key feature of the water-water cycle is that it uses water as an electron donor and produces water as an end product, preventing the accumulation of excess reducing agents without the synthesis of ATP or NADPH. Under stress conditions, when light intensity exceeds the capacity of photochemical quenching or when the activity of the Calvin cycle is reduced, the plastoquinone pool is over-reduced, and ^3^Chl accumulates, leading to an explosive increase in ^1^O_2_ and O_2_·^−^ production [25]. Despite the presence of a powerful antioxidant system, including carotenoids (^1^O_2_ quenchers), tocopherols, and enzymes, chronic stress can deplete these pools, leading to oxidative damage to the D1 and D2 proteins of the PSII reaction center, lipid peroxidation of thylakoid membranes, and, ultimately, photoinhibition [28]. Thus, chloroplasts act not only as energy sources but also as specific sensors that convert the effect of the stressor into various signaling pathways (retrograde signaling), including the production of reactive carbonyl, nitrogen, and sulfur compounds, as well as precursors of stress hormones [28].

### 2.3. Regulation of ROS in Mitochondria: Energy Balance and Signaling

Although chloroplasts dominate ROS production in green tissues exposed to light, mitochondria are the primary generators of reactive oxygen species in non-photosynthetic tissues (roots, tubers) and in the dark [29]. The main sites of electron leakage in the mitochondrial ETC are Complex I (NADH dehydrogenase) and Complex III (ubiquinol-cytochrome c reductase). Here, one-electron reduction of oxygen occurs, forming O_2_·^−^, which is then dismutated into H_2_O_2_ by superoxide dismutase MnSOD in the matrix and Cu/ZnSOD in the intermembrane space (Figure 1B) [30]. The current model of the respiratory chain suggests that complexes I, III, and IV form supercomplexes (respirasomes) that ensure efficient electron transfer with minimal leakage to oxygen [31]. Disruption of the assembly of these supercomplexes under stress leads to increased O_2_·^−^ formation. In contrast to previous concepts, mitochondrial ROS are now considered not simply random “leaks,” but important mediators of physiological cellular signaling, informing the nucleus about the cell’s energy status (mitochondrial retrograde regulation) [32].

To control ROS homeostasis, mitochondria possess a specialized defense system. A key role is played by alternative oxidase (AOX), which catalyzes the transfer of electrons from ubiquinol to oxygen, bypassing Complexes III and IV. This “shortcut” reduces the overreduction of the ubiquinone pool, thereby preventing O_2_·^−^ formation [32]. Studies on *Arabidopsis* mutants *aox1a* lacking AOX have shown increased sensitivity to combined drought and high light stress, confirming the critical role of AOX in oxidative stress protection [33].

In addition to AOX, MnSOD functions in the mitochondria, converting O_2_·^−^ into H_2_O_2_, which is then reduced to water by ascorbate peroxidase (APX) or enzymes of the ascorbate-glutathione cycle [34]. The balance between ROS generation in the respiratory chain and their neutralization is particularly important under abiotic stresses such as high temperatures or drought. Under these conditions, the respiration rate increases to compensate for the deficiency of ATP produced by chloroplasts, which increases the load on the respiratory chain [35].

### 2.4. ROS Regulation in Peroxisomes: A Metabolic Hub of Oxidative Stress

Plant peroxisomes are highly dynamic membrane-bound organelles characterized by adaptive nitroxidative metabolism [36,37]. Unlike chloroplasts and mitochondria, where ROS are a byproduct of electron transfer, in peroxisomes, the production of H_2_O_2_ and O_2_·^−^ is an integral part of catabolic processes (Figure 1C). The main enzyme generating H_2_O_2_ in peroxisomes is glycolate oxidase (GOX), which is involved in photorespiration [38]. In addition, fatty acid β-oxidation oxidases, flavin oxidases, and xanthine oxidase (XOD) make significant contributions. XOD generates O_2_·^−^ in the peroxisomal matrix by converting xanthine and hypoxanthine to uric acid, using NADH and cytochrome b as electron acceptors [39]. The O_2_·^−^ produced is then dismutated to H_2_O_2_ by peroxisomal SOD (e.g., Cu/Zn-SOD/SOD1) [40,41]. In parallel, urate oxidase (UOX/uricase) contributes to the peroxisomal H_2_O_2_ pool through a distinct reaction: it catalyzes the oxidation of uric acid by molecular oxygen to 5-hydroxyisourate, releasing H_2_O_2_ as a by-product. Thus, UOX is not a superoxide dismutase; its role in peroxide metabolism is catalytic generation of H_2_O_2_ from uric acid, which is functionally separate from the SOD-mediated dismutation of O_2_·^−^ [40,41].

It is worth noting that the photorespiratory pathway, which involves peroxisomes, is a colossal source of H_2_O_2_. When stomata close (e.g., in response to drought or ABA), the CO_2_ concentration inside the leaf drops and O_2_ rises, sharply increasing the oxygenase activity of Rubisco and, consequently, H_2_O_2_ production by glycolate oxidase. This makes peroxisomes key players in the signaling pathways regulating stomatal conductance [38,42].

To maintain homeostasis, peroxisomes possess a powerful detoxification system. Catalase (CAT) is the main enzyme that degrades H_2_O_2_ without consuming reducing agents. In addition to CAT, peroxisomes contain APX and a full complement of components of the ascorbate-glutathione cycle (AsA-GSH). Decreased activity of this cycle or ascorbate levels leads to lipid peroxidation in peroxisomal membranes, indicating the critical importance of the AsA-GSH pool for the structural integrity of organelles [43]. Furthermore, peroxisomes host polyamine oxidases (POXs), which are involved in the catabolism of polyamines, generating H_2_O_2_ as a byproduct. This process links redox signaling to the regulation of gene expression responsible for ROS generation and removal [11,44].

### 2.5. ROS Regulation in the Apoplast: A Frontier of Defense and Signaling

The apoplast plays a special role in ROS generation, as ROS are formed here outside the cell and serve as primary signals for intercellular communication and pathogen response. The main generators of O_2_·^−^ in the apoplast are the NADPH oxidases of the respiratory burst (Respiratory Burst Oxidase Homologs, Rboh). In *Arabidopsis*, particularly in stomatal guard cells, the *AtRbohD* and *AtRbohF* genes play a key role [41]. RBOH transfer electrons from cytosolic NADPH to extracellular oxygen, generating O_2_·^−^, which is then dismutated into H_2_O_2_ by the action of apoplastic SOD. In addition to NADPH oxidases, cell wall peroxidases (class III), polyamine oxidases, and oxalate oxidases contribute to the apoplastic H_2_O_2_ pool. Oxalate oxidase catalyzes the formation of H_2_O_2_ and CO_2_ from oxalic acid, which is particularly important in defense against fungal infection [34,45]. Amine oxidases catalyze the oxidation of biogenic amines to aldehydes with the release of NH_3_ and H_2_O_2_. The accumulation of polyamines (putrescine) during hypoxia or anoxia in rice and wheat shoots, along with the predominant localization of amine oxidase in the apoplast, indicates the involvement of these enzymes in H_2_O_2_ generation during oxygen starvation [16].

Apoplastic H_2_O_2_ is a key second messenger. An increase in its level leads to the opening of calcium channels, an increase in the Ca^2+^ concentration in the cytosol, and the activation of Ca^2+^-dependent protein kinases (CDPK). This triggers a cascade of reactions including the synthesis of polyamines and ABA, which forms a positive feedback loop that amplifies the stress signal [44,46]. Thus, apoplastic ROS do not so much damage the cell as coordinate systemic acquired immunity (SAR) and acclimation to abiotic stress.

### 2.6. Antioxidant Network: Balance of ROS Generation and Scavenging

Abiotic stress factors (drought, salinity, extreme temperatures, heavy metals) cause an imbalance between ROS generation and detoxification, leading to oxidative stress [16]. To combat the effects of stress, plants have developed a multi-level antioxidant defense system.

The enzymatic system includes: First line of defense: Superoxide dismutase (SOD), Catalase (CAT), Ascorbate peroxidase (APX). Redox buffer regeneration: Glutathione reductase (GR), Monodehydroascorbate reductase (MDHAR), Dehydroascorbate reductase (DHAR). Accessory enzymes: Glutathione peroxidase (GPX), Guaiacol peroxidase (GOPX), Glutathione-S-transferase (GST) [47]. The non-enzymatic system includes low-molecular antioxidants: ascorbic acid (AsA), glutathione (GSH), phenolic acids, alkaloids, flavonoids, carotenoids, α-tocopherol, and non-protein amino acids [47].

Normally, the antioxidant system maintains a stable redox equilibrium necessary for the regulation of growth, development, and programmed cell death [14,48]. However, under severe stress, excessive ROS generation overloads defense mechanisms, causing oxidative damage to lipids (peroxidation), proteins (carbonylation), and DNA, leading to metabolic disorders and, ultimately, cell death [48,49].

It is important to emphasize that plant hormones are closely linked to the regulation of redox homeostasis. They modulate the activity of antioxidant enzymes and promote the accumulation of low-molecular-weight antioxidants, increasing stress resistance [50,51]. The interaction between ROS, antioxidants, and phytohormones determines plant survival under stressful conditions.

## 3. NADPH Oxidases (RBOH): Central Generators of Apoplastic ROS

### 3.1. Structure and Diversity of Plant RBOH

The apoplast is the compartment in direct contact with the environment, and therefore its composition provides the first level of plant defense against pathogens and various abiotic stressors [52]. Apoplast proteins and oligosaccharides are involved in the initial perception of external signals and facilitate the generation and transmission of signals over long distances. Under conditions of abiotic stress, reactive oxygen species (ROS) are produced in the plasma membrane and apoplast, triggering cascades of protective reactions. Among the enzymes responsible for ROS generation in the apoplast (NADPH oxidases NOX/RBOH, amine oxidases, polyamine oxidases, oxalate oxidases, and class III peroxidases), NADPH oxidases, known in plants as RBOH (respiratory burst oxidase homologs), are considered the most important enzymes generating ROS in response to hormonal signals and environmental stimuli [53]. They catalyze the transfer of electrons from cytosolic NADPH to extracellular oxygen to form O_2_·^−^, which can then be dismutated to H_2_O_2_, a stable signaling molecule. Plant RBOH proteins are integral plasma membrane proteins containing six transmembrane domains (Figure 2). The third and fifth transmembrane domains each contain two pairs of histidine residues that coordinate two heme groups, which are necessary for electron transfer across the membrane. At the C-terminus of the protein are cytosolic domains that bind FAD (flavin adenine dinucleotide) and NADPH (nicotinamide adenine dinucleotide phosphate), which mediate catalytic activity [54]. The N-terminal region (approximately 300 amino acids) contains two calcium-binding EF-hand motifs and several phosphorylation sites, which play a key role in regulating enzyme activity [54].

RBOHs are represented by multigene families in all studied plant species [55,56]. The diversity of RDOH oxidase indicates the functional specialization of individual isoforms in different tissues and processes. RBOHs are considered central nodes of the reactive oxygen species signaling network and play important roles in stress responses and plant development [57]. RBOHs have been shown to be involved in pollen development, pollen tube growth, root hair formation, seed germination, plant-microbial interactions, and immunity [55,58].

### 3.2. Molecular Mechanisms Regulating RBOH Activity

RBOH protein activity is regulated through complex mechanisms involving Ca^2+^ binding, phosphorylation, interactions with lipid mediators, and the formation of positive feedback loops involving ROS and Ca^2+^ (Figure 3) [59,60].

**The Role of Phosphorylation**. Modification of RBOH proteins by phosphorylation is crucial for the regulation of their activity and for signal transduction during plant development and adaptation to the environment. Phosphorylation of RBOH proteins is regulated by protein kinases and protein phosphatases, primarily at serine (Ser), threonine (Thr), and tyrosine (Tyr) residues [61]. Precise phosphorylation of proteins at specific sites and by specific kinases determines their properties [62], which is important for a variety of signaling pathways triggered either by intrinsic phytohormonal signals or external environmental factors [63].

**Ca^2+^-dependent activation.** It has been suggested that RBOHC phosphorylation may act in concert with Ca^2+^ oscillations. All plant RBOHs contain EF-hand motifs, indicating that their activation is obligately dependent on Ca^2+^ ions. RBOHD involvement in the fast systemic signaling pathway is H_2_O_2_-dependent and requires phosphorylation by multiple Ca^2+^-dependent and Ca^2+^-independent kinases, including CIPK and CPK family members, as well as BIK1 and OST1/SnRK2. CIPK is thus one important component of a broader kinase network rather than the exclusive regulator of RBOHD in systemic signaling. This multi-kinase control supports the hypothesis that Ca^2+^-dependent ROS production is involved in long-range ROS propagation, while allowing stimulus-specific tuning of RBOHD activity [64]. RBOHF activity is also regulated by direct Ca^2+^ binding to EF-hand motifs and Ca^2+^-dependent phosphorylation by the CBL–CIPK complexes [59]. Structural analysis of OsRbohB from rice showed that Ca^2+^ directly binds only to the first EF-hand motif but not to the second, indicating fine-tuning of calcium sensitivity [65]. Thus, different RBOH isoforms may have specific regulatory mechanisms, allowing for fine-tuning of responses to a variety of stimuli.

**Positive feedback between Ca^2+^ and ROS**. For RBOHD and RBOHF, Ca^2+^ binding and phosphorylation act synergistically to activate ROS production [66,67]. An increase in cytosolic Ca^2+^ concentration is required for RBOHD activation, and this activation requires conformational changes in the EF-hand motifs upon Ca^2+^ binding [66]. ROS generation at the plasma membrane leads to the release of H_2_O_2_ into the apoplast, where it activates calcium channels, stimulating Ca^2+^ influx into the cytosol. This causes a second increase in Ca^2+^ levels, which again activates RBOH, closing the positive feedback loop [54,68]. This mechanism ensures amplification and maintenance of the signal, which is particularly important during polar cell development and the response to hypoxia [65,69]. OsRbohB-dependent ROS production has been shown to be required for Ca^2+^ influx, and induced ROS, in turn, cause Ca^2+^ mobilization from intracellular stores [65]. Similar results were obtained in other systems, which indicates the universality of this mechanism [70].

### 3.3. Involvement of RBOH in the Response to Abiotic Stresses

RBOHs play an important role in plant responses to various abiotic stresses, including drought, salinity, extreme temperatures, and hypoxia [41,57].

**Salt Stress**. Salt stress causes ionic and osmotic damage, slows plant growth, and reduces yield. Maintaining cellular Na^+^/K^+^ homeostasis is critical for survival in saline soils [71]. In Arabidopsis, AtRBOHD and AtRBOHF produce ROS that function as signaling molecules regulating the Na^+^/K^+^ balance, thereby enhancing salt tolerance [72]. Double mutants *atrbohD1/F*1 and *atrbohD2/F2* exhibit increased sensitivity to NaCl and less efficient selective K^+^ uptake compared to wild-type plants [72,73]. Early H_2_O_2_ formation under salt stress triggers antioxidant responses that prevent excessive ROS accumulation and mitigate oxidative damage. *AtRBOHD* and *AtRBOHF* expression is significantly induced by salinity, with AtRBOHF involved in regulating Na^+^ loading into xylem, protecting leaves from the toxic effects of salt [73]. Salt-induced increases in cytosolic Ca^2+^ concentration are associated with Ca^2+^ influx through the plasma membrane and release from intracellular stores, which activates NOX/RBOH and leads to apoplastic accumulation of H_2_O_2_ [73]. Furthermore, AtRBOH-dependent H_2_O_2_ production promotes the accumulation of proline, an osmoprotectant that increases salt tolerance [74,75].

**Hypoxic stress**. Flooding and the resulting hypoxia (oxygen deficiency) severely impair plant energy metabolism. Plants have adaptive mechanisms triggered by changes in the redox state and phytohormonal balance [76]. In Arabidopsis, hypoxia increases transcript levels of five RBOH genes—*AtRBOHA*, *B*, *D*, *G*, and *I*—with *AtRBOHD* expression significantly increasing early in the response. Ethylene modulates H_2_O_2_ signaling by regulating *RBOH* gene expression in the hypoxic pathway [77].

AtRBOHI is also induced by hypoxia and regulates the expression of genes involved in ethylene biosynthesis and downstream hypoxic signaling pathways [76]. In rice, differential expression of NOX/RBOH genes is observed during water submersion, which maintains ROS homeostasis and helps seedlings adapt to varying degrees of flooding [78]. In particular, *OsRBOHH* expression under oxygen-deficient conditions is higher in root cortical cells than in other tissues. ROS production by OsRBOHH, induced by CDPK5 and/or CDPK13 kinases, is necessary for aerenchyma formation in rice roots exposed to ethylene during hypoxia [78].

**Temperature Stress**. Numerous studies show that NOX/RBOH respond to cold and heat stress. In maize, NADPH oxidase activity increases after 2 h of chilling in seedlings but returns to control levels after 24 h [79]. In Arabidopsis, cold exposure induces AtSRC2 gene expression in roots, which is proportional to the level of ROS, partly produced by AtRBOHF. AtSRC2 protein is an activator of Ca^2+^-dependent ROS production mediated by AtRbohF and plays a role in the cold response [80]. Similarly, in strawberry, *FvRBOHA* and *FvRBOHD* expression levels rapidly increase upon cold exposure, accompanied by an increase in NADPH oxidase activity [81]. In citrus fruits, cold induces the expression of *CsRBOHD*, *CsRBOHE*, and *CsRBOHF*, and suppression of *CsRBOHD* leads to increased cold tolerance, indicating the involvement of the CsRBOHD-ROS signaling pathway in the induction of antioxidant defense genes [82].

At high temperature (30 °C), total NADPH oxidase activity and *NtRBOHD* expression were suppressed in tobacco leaves infected with tobacco mosaic virus (TMV), resulting in reduced ROS production, increased susceptibility to pathogens, and suppression of stress-induced necrosis [81]. This suggests the importance of RBOH in thermotolerance and the immune response.

**Drought**. NADPH oxidases are involved in ABA-induced ROS production, which triggers adaptive responses to water stress. In maize, water stress causes ABA accumulation, leading to NOX/RBOH activation and ROS generation, which in turn activates the antioxidant defense system against oxidative damage [73]. In rice, drought increases overall NADPH oxidase activity, and *OsRBOHA* expression is stimulated under water stress conditions. Deletion of *OsRBOHA* reduces plant tolerance to water stress, while overexpression increases tolerance, making this gene promising for genetic engineering [83].

Interesting data regarding the role of RBOH under drought conditions have been obtained from various cultivars of *Festuca arundinacea*. A drought-sensitive cultivar of this fescue grass exhibited elevated levels of FRBOHD transcripts and higher H_2_O_2_ content in its leaves, whereas a drought-tolerant cultivar showed lower levels. This suggests that the tolerant cultivar maintains a moderate level of ROS production, while the sensitive cultivar experiences excessive ROS production, which may exacerbate damage to the plant’s leaves [84]. Thus, the precise fine-tuning of RBOH activity is crucial for maintaining a balance between protective signaling and oxidative stress.

## 4. The Dual Nature of ROS: The ROS Signal Wave

According to current concepts, two main sources of ROS are formed in plant cells during abiotic stress [85]. The first, metabolic ROS, are generated as a result of disruptions in normal metabolic processes, primarily in the electron transport chains of chloroplasts, mitochondria, and peroxisomes. Their accumulation reflects a disruption in cellular homeostasis and can directly affect the redox status of key enzymes that limit the rate of metabolic fluxes. This, in turn, allows the cell to quickly restructure metabolism in response to stress [86]. Furthermore, metabolic ROS can alter the functions of regulatory proteins through reversible oxidative modifications, affecting transcription and translation.

The second pool, signaling ROS, is produced in a specialized manner as part of an active response to stress perception. Their generation is mediated by stress sensors, such as heat-activated cyclic nucleotide channels, and then by NADPH oxidases of the RBOH (Respiratory Burst Oxidase Homolog) family on the plasma membrane. RBOH activation occurs through increased cytosolic Ca^2+^ levels and/or phosphorylation, which couples ROS signals to calcium and protein kinase cascades [87,88].

Importantly, these two ROS pools are not isolated. Metabolic ROS (e.g., in chloroplasts) and signaling ROS (in the apoplast) can influence each other’s levels and even shuttle between compartments. In particular, H_2_O_2_ is sufficiently stable and penetrates membranes, including in a regulated manner via aquaporins, which allows it to act as an intercellular and interorgan signal [89]. Thus, compartmentalization of ROS does not interfere with their systemic action, but rather determines the specificity of the initial signal and its modulation.

### 4.1. Systemic Signaling: Rapid Preparation for Stress

From an evolutionary perspective, the ability to rapidly notify all parts of a plant of local stress represents a powerful adaptation. Pathogens require hours to disseminate from infected tissue, and abiotic damage often accumulates gradually, allowing time for the systemic signal to unfold [90]. Plants utilize a variety of systemic signals, which can be categorized by their rate of dissemination. Slow systemic signals (lasting from hours to days) include hormones (abscisic acid, jasmonate, salicylate), metabolites, and some proteins. They mediate long-term restructuring of metabolism and gene expression. In contrast, fast systemic signals (seconds to minutes) include electrical signals, Ca^2+^ waves, hydraulic changes, and, the focus of this review, the ROS wave [91]. These rapid signals are required to trigger the so-called systemic acquired adaptation (SAA), which allows uninjured organs to increase their resistance to future stress [27,33].

Systemic signals can propagate via both the symplastic (plasmodesmata) and apoplastic (extracellular) pathways. Phloem has been shown to transport signaling molecules—from proteins and mRNA to small metabolites—at speeds on the order of hundreds of micrometers per second [92]. However, the ROS wave, as a self-sustaining process, can reach speeds comparable to electrical signals and spread across various tissues [93].

### 4.2. The ROS Wave: Molecular Mechanism, Propagation, and Regulation

The ROS wave is a unique intercellular signaling process based on the autocatalytic generation of ROS by neighboring cells (Figure 4). The key protein mediating this process is RBOHD [94,95]. The mechanism consists of the following steps: Local stress activates RBOHD in the cell, which triggers the release of ROS (mainly H_2_O_2_ and O_2_·^−^) into the apoplast. Neighboring cell perception: ROS accumulates in the apoplast and is sensed by receptors or sensors on the plasma membrane of neighboring cells [96]. Amplification: This perception leads to activation of the neighboring cell’s own RBOHD, often through a Ca^2+^-dependent pathway and phosphorylation, which generates a new batch of ROS. Spreading: The cycle repeats, creating a self-sustaining wave that can traverse the entire plant.

Initially, the ROS wave was monitored using reporter constructs (e.g., Zat12 or WRKY40 promoters driving luciferase [27,97]. Recent advances in whole-plant imaging have allowed this wave to be observed in real time in living soil plants [93]. These studies confirmed that the ROS wave is induced by a variety of stimuli—heat, light stress, wounding, or pathogens—and is suppressed in *rbohD* mutants. The inhibitor diphenyleneodontium (DPI), which blocks NADPH oxidases, as well as exogenous catalase, which degrades H_2_O_2_, effectively stop the propagation of the wave [27,97]. Interestingly, the tissue pathway of the ROS wave depends on the type of stress. Under light stress, the wave propagates predominantly through avascular tissue (mesophyll and epidermis), while under other stresses, it propagates through the vascular system [93]. Moreover, different tissues can interact: for example, vascular tissue is involved in the systemic stomatal response to light stress, as shown [98]. This indicates the plasticity of propagation pathways and the integration of the ROS wave with the anatomical features of the plant.

### 4.3. The ROS Wave as a Trigger of Systemic Adaptation and a Coordinator of Stomatal Movements

The ROS wave is considered a universal alarm signal that does not carry specific information about the type of stressor, but prepares tissues for general acclimation rearrangements. It is believed that it activates a general acclimation program, leading to widespread transcriptomic and metabolomic reprogramming [27,95]. It is the presence of the ROS wave, rather than its specific features, that determines the possibility of systemic acclimation. However, in the absence of the wave (e.g., in *rbohD* mutants), systemic defenses do not develop, which emphasizes its critical role.

Recently, a new function of the ROS wave has been identified: coordination of systemic stomatal behavior [97,98]. Devireddy et al. [97] showed that in response to local light stress, the ROS wave induces systemic stomatal closure throughout the plant. Conversely, under heat stress, the same wave coordinates the opening or modulation of stomatal conductance to optimize cooling [98]. This demonstrates that the same signal can trigger different physiological responses depending on the context and interactions with other regulatory networks (e.g., hormonal ones). Control of stomatal conductance affects not only water balance and gas exchange but also hydraulic waves, creating feedback loops between signaling systems.

### 4.4. Subcellular Localization, Tissues, and Intersystemic Interactions

One of the most important questions remains: through which cell layers and tissues do systemic signals propagate, and what is the role of subcellular localization? There is evidence that specific plastids—the so-called “sensor plastids” in the epidermis and vascular parenchyma—are involved in the perception of initial stress [99]. Moreover, different signals prefer different routes: ROS and ATP act in the apoplast, Ca^2+^ and some metabolites are transmitted symplastically through plasmodesmata, while electrical signals are associated with changes in membrane potential. Critically, these systems are not isolated. ROS waves can influence calcium waves by altering channel permeability, and vice versa—Ca^2+^ activates RBOHD [33]. Electrical signals, in turn, can modulate the membrane potential and enhance or attenuate ROS generation.

Thus, the systemic response is an integrated network in which the ROS wave is one thread intertwined with calcium and electrical signals, as well as hormonal cascades. This intertwining ensures both the speed and specificity of the response. This integration may explain why different stressors that induce the same ROS wave lead to different phenotypic outcomes.

### 4.5. Biphasic Nature of ROS Production: Relationship to the Wave

Many stress responses in plants are accompanied by a biphasic surge of ROS: the first peak occurs within minutes, the second—hours or days later [94,100,101]. An early O_2_·^−^ surge has been shown to occur as early as 3 min after mechanical injury, with secondary O_2_·^−^ and H_2_O_2_ production observed after 6 h. Inhibition of early ROS generation with DPI prevents the late phase, suggesting a causal relationship [101].

Current evidence suggests that the ROS surge connects these two phases, transmitting a signal from local tissue (where the initial surge occurs) to systemic tissues, where it triggers secondary changes, including reprogramming of gene expression and activation of protective proteins [88,94]. Thus, the ROS surge is not only a rapid systemic signal but also a mediator that mediates the transition from an acute response to long-term adaptation.

### 4.6. ROS Sensors and Receptors: Oxidative Post-Translational Modifications

The signaling function of ROS, particularly H_2_O_2_, is largely mediated by oxidative post-translational modifications (oxy-PTMs) of proteins. Unlike classical hormones, which act through receptor binding, ROS act directly on chemically sensitive moieties, primarily the thiol (–SH) groups of cysteine residues. These moieties are oxidized by H_2_O_2_ to sulfenic acid (–SOH), which is highly reactive and reversible [102]. Further oxidation to sulfinic (–SO_2_H) or sulfonic (–SO_3_H) acids is usually irreversible and is associated with loss of protein function [48].

Furthermore, H_2_O_2_ can induce the formation of both intramolecular and intermolecular disulfide bonds, which alter protein conformation, its activity (including enzymatic and transcriptional), subcellular localization, and interactions with partners [103,104,105]. Sulfenic acid reduction is mediated by the glutaredoxin (GRX), peroxiredoxin (PrxR), and thioredoxin (TRX) system, which plays a key role in dynamic signal regulation.

### 4.7. Key Sensor Proteins: HPCA1, GPX3, and Others

One of the best-studied sensors of extracellular H_2_O_2_ is the transmembrane kinase HPCA1 (HYDROGEN-PEROXIDE-INDUCED Ca^2+^ INCREASES 1), which contains leucine-rich repeats. Upon oxidation of H_2_O_2_, HPCA1 forms two intermolecular disulfide bridges, which facilitate Ca^2+^ entry into the cell and activate calcium signaling. This allows HPCA1 to play a central role in cell-to-cell ROS signaling, systemic signaling during abiotic and biotic stress, and local and systemic responses [93,106]. HPCA1’s high sensitivity to oxidation makes it an ideal “receiver” of the ROS wave. Another important sensor is the cytosolic GPX3 (glutathione peroxidase-like protein), which, by absorbing H_2_O_2_, alters its redox status and influences the activity of the phosphatase ABI2 (ABA INSENSITIVE 2). ABI2 is a negative regulator of the abscisic acid (ABA) signaling pathway and stomatal closure. Thus, GPX3 acts as an oxidative-sensitive modulator of the hormonal signal, integrating the ROS signal with the ABA pathway [107]. Another example is the redox regulation of chloroplast enzymes through the ferredoxin-thioredoxin reductase (FTR/TRX) and thioredoxin-like protein 2 (TRXL2)/2-Cys peroxiredoxin cascades, which enables rapid adaptation of the photosynthetic apparatus to changes in light [108].

These examples demonstrate that ROS, acting through oxy-PTMs, can alter the activity of not only individual enzymes but also entire regulatory networks, enabling their involvement in fine-tuning stress responses.

## 5. Ca^2+^ as a Second Messenger: From Signature to Response

### 5.1. The Concept of Ca^2+^ Signatures

The concentration of free Ca^2+^ in the cytosol of resting cells is maintained at an extremely low level (on the order of 10^−7^ M), which is approximately four orders of magnitude lower than in the extracellular environment (10^−3^ M) [109]. This enormous electrochemical gradient creates the preconditions for a rapid and significant increase in the cytosolic Ca^2+^ concentration ([Ca^2+^]_cyt_) upon the opening of Ca^2+^-permeable channels in response to various stimuli. It is this ability to generate fast, high-amplitude, and spatially organized [Ca^2+^]_cyt_ transients that underlies the signaling function of Ca^2+^ [110]. Temporal and spatial changes in Ca^2+^ concentrations in the cytoplasm and organelles that occur in response to specific stimuli are called Ca^2+^ signatures [111]. These signatures are characterized by a specific amplitude, frequency, duration, and spatial organization, which allows them to encode information about the nature, intensity, and duration of the stress exposure. Ca^2+^ signatures represent the first level of specificity in the Ca^2+^ signaling process, while Ca^2+^ sensors and their targets provide the second level of specificity [112,113].

Plant perception of abiotic stress triggers a cascade of events, the first of which is a change in the level of free Ca^2+^ in the cytosol. These changes are temporal and spatial in nature, specific to each type of stimulus, and are referred to as Ca^2+^ signatures [111,114]. Ca^2+^ signatures encode information about the nature of the stressor, its intensity, and duration through variations in the amplitude, frequency of oscillations, duration of individual transients, and subcellular localization of the signal.

Cellular processes are associated with spatial and temporal changes in Ca^2+^ levels in the cytoplasm or organelles. Transient changes in Ca^2+^ concentration during growth and development, as well as in response to stress signals, are characteristic of a particular stimulus. For example, various abiotic stresses—drought, salinity, heat, or cold stress—induce characteristic Ca^2+^ signatures that differ in amplitude and kinetics.

Ca^2+^’s ability to serve as a signaling molecule is due to several unique properties. First, the enormous Ca^2+^ concentration gradient across membranes allows for rapid changes in [Ca^2+^]_cyt_. Secondly, the chemical properties of Ca^2+^, including the flexibility of coordination with carboxyl and neutral oxygen groups in proteins, make it uniquely suited for interaction with biologically active molecules. Thirdly, a rapid rise in Ca^2+^ levels against a background of low concentrations creates a signal easily distinguishable from noise. Finally, the toxicity of elevated Ca^2+^ concentrations requires the rapid activation of homeostasis restoration mechanisms, which include the coordinated action of Ca^2+^ transporters and sensor molecules—an evolutionarily evolved signaling toolkit.

### 5.2. Ca^2+^ Homeostasis: Channels, Pumps, and Exchangers

Maintaining low [Ca^2+^]_cyt_ levels and generating Ca^2+^ signals are ensured by a complex system of membrane transporters, including three main categories: Ca^2+^ channels, which provide passive influx of Ca^2+^ into the cytosol along an electrochemical gradient. These include hyperpolarization-activated channels, depolarization-activated channels, and voltage-independent channels [115]. Ca^2+^ channels can be rapidly activated (with a total activation time in milliseconds), slowly activated (with a total activation time in seconds), or operate as single channels. Channel activity is regulated by modulators such as cytosolic Ca^2+^, H^+^, reactive oxygen species, and polyamines. Ca^2+^-ATPases and Ca^2+^/H^+^ exchangers transport Ca^2+^ against the electrochemical gradient, ensuring the efflux of Ca^2+^ from the cytosol to the apoplast or intracellular stores [115,116].

The main Ca^2+^ reservoirs in plant cells are vacuoles, the endoplasmic reticulum (ER), mitochondria, plastids, and the apoplast [117]. Membrane channels regulate the influx of Ca^2+^ from these stores into the cytosol [118,119]. Most plant Ca^2+^ channels are non-selective cation channels (NSCCs), which are highly permeable to mono- and divalent cations (K^+^, Na^+^, NH_4_^+^, Cs^+^, Rb^+^, Ba^2+^, Sr^2+^) and weakly permeable to certain ions, such as Zn^2+^, Mn^2+^, and Mg^2+^ [120]. Five major families of Ca^2+^ channels regulating ion influx have been identified in Arabidopsis: cyclic nucleotide-gated channels (CNGCs), glutamate-like receptors (GLRs), two-pore channels (TPCs), mechanosensitive channels, and channels induced by decreased hyperosmolality and increasing [Ca^2+^]_cyt_ (OSCAs) [118,119].

### 5.3. Plant Ca^2+^ Sensors: Structure, Function, and Specificity

Ca^2+^ signals are perceived by specialized Ca^2+^ sensors—proteins that bind to Ca^2+^ and change their conformation and function upon binding. These sensors transmit the signal to downstream components, triggering phosphorylation cascades, changes in gene expression, and physiological responses. Four main families of Ca^2+^ sensors have been identified in plants, most of which contain EF-hand domains—helix-loop-helix structural motifs that form a pocket for Ca^2+^ binding [120].

**Calmodulins (CaM) and CaM-like Proteins (CML).** Calmodulin (CaM) is the most conserved Ca^2+^-binding protein found in all eukaryotes. CaM consists of two pairs of EF-hands, each formed by a helix-loop-helix motif. In each pair, the two EF-hands are connected by an α-helix; each pair of EF-hands forms a globular structure, so that the entire CaM protein consists of two globular domains. Upon binding Ca^2+^, CaM’s conformation changes, exposing hydrophobic surfaces for binding to its targets [121,122]. CML (CaM-like proteins) are found only in plants and differ from CaM in sequence, Ca^2+^-binding properties, and target spectrum. Sequence differences between CaM and CML reflect their distinct Ca^2+^ binding properties and their specific targets. CaM/CML targets include promoters, enzymes (including kinases and phosphatases), transcription factors, and ion channels. Some CaMs are involved in abiotic stress, while others are involved in biotic stress [121,122]. CaM and CMLs belong to the “sensor-relay” group because they transmit signals through conformational changes and interactions with effector proteins.

**Calcineurin B-like proteins (CBLs) and CBL-interacting protein kinases (CIPKs).** CBLs are another family of Ca^2+^ sensors unique to plants. Unlike CaMs, CBLs do not possess their own enzymatic activity but function through interactions with CBL-interacting protein kinases (CIPKs) [123]. CBL-CIPK complexes form biomolecular sensor modules that regulate a variety of physiological processes, including ion transport and stress responses [123]. The CBL-CIPK system plays a key role in regulating ion transport in response to stress. Like CML, CBL belongs to the “sensor-relay” group.

**Ca^2+^-Dependent Protein Kinases (CDPK/CPK).** CDPKs (also referred to as CPKs) are a unique family of Ca^2+^ sensors in which the Ca^2+^-binding domain and effector (kinase) domain are combined in a single polypeptide chain [124]. This allows CDPKs to directly translate changes in Ca^2+^ concentration into phosphorylation of target proteins without the involvement of additional intermediaries. Due to this feature, CDPKs are classified as “sensor-responders” [125]. CDPKs are found in plants and protozoa, but are absent from animals. They are involved in the regulation of a wide range of stress responses, including responses to drought, salinity, heat, and cold stress.

**Ca^2+^/CaM-dependent protein kinases (CCaMKs).** CCaMKs are another type of Ca^2+^ sensor, unique to plants. They contain both a CaM-binding domain and a kinase domain, allowing them to integrate Ca^2+^ and CaM-dependent signals. CCaMKs are involved in the regulation of a number of stress responses and also play an important role in symbiotic interactions between plants and microorganisms [126,127].

### 5.4. Generation and Propagation of Ca^2+^ Signals: Channels and Waves

#### 5.4.1. Ca^2+^-Permeable Channels: From Local Flux to Systemic Wave

Ca^2+^ influx into the cytosol under stress is mediated by several families of Ca^2+^-permeable channels located on the plasma membrane and membranes of intracellular organelles [114,118].

**CNGCs** (Cyclic Nucleotide-Gated Channels) are channels activated by cyclic nucleotides. They are involved in a variety of signaling pathways, including responses to abiotic and biotic stress. CNGCs are non-selective cation channels permeable to Ca^2+^ [128].

**GLRs** (Glutamate Receptor-Like Channels) are channels homologous to the ionotropic glutamate receptors of animals. In Arabidopsis, the GLR family contains 20 members. GLRs appear to be closely linked to fast, long-range electrical signaling [128]. Recent studies have shown that glutathione (GSH) acts as a critical signaling molecule that utilizes GLR3.3 to trigger long-range Ca^2+^-dependent defense signals [129].

**TPC1** (Two Pore Channel 1) is a vacuolar two-pore channel that plays a key role in Ca^2+^-induced Ca^2+^ release (CICR) from vacuoles [130]. TPC1 contains two classical Ca^2+^-binding EF-hand domains on its cytosolic surface (although only one may be functional upon Ca^2+^ activation). TPC1 exemplifies the complex array of potential interactions that contribute to systemic signal propagation [131].

**OSCA** (Reduced Hyperosmolarity-induced [Ca^2+^]_cyt_ Increase channels) are channels activated by decreased osmolarity and are presumably mechanosensitive. They are involved in the perception of osmotic stress [132].

**Annexins** (ANNs) are also considered potential Ca^2+^ channels or their regulators, sensitive to ROS [133].

#### 5.4.2. The Ca^2+^ Wave Phenomenon: Systemic Signal Propagation

Fluctuations in cytosolic calcium levels in response to external stimuli, propagating from the site of perception to distant tissues, are known as calcium waves [134]. This phenomenon represents a fundamental systemic signaling mechanism that allows plants to coordinate responses to local stressors. It was initially thought that systemic propagation of the Ca^2+^ signal could be mediated by direct Ca^2+^ movement along the xylem [33]. However, evidence has subsequently been obtained for a more complex mechanism—cell-to-cell propagation of increased cytosolic Ca^2+^ levels, termed the Ca^2+^ wave. This Ca^2+^ wave links local perception of stimuli, such as injury or salt stress, with adaptive responses throughout the plant. The Ca^2+^ wave induced by salt stress propagates at a velocity significantly different from that of the ROS wave [134]. While the injury-induced ROS wave propagates at ~1400 μm s^−1^ through vascular tissue, the Ca^2+^ wave appears to propagate more slowly and is confined to the cortex and endoderm [90]. This difference may be due to the fact that the ROS wave propagates through the vascular system, which is well-adapted for long-distance signaling.

Importantly, the propagation of the Ca^2+^ wave induced by both salt stress and injury is dependent on TPC1 (Figure 5) [135]. This suggests the presence of conserved elements of the signal propagation system common to different types of stress.

The key mechanism underlying the propagation of the Ca^2+^ wave is Ca^2+^-induced Ca^2+^ release (CICR). According to this model, an increase in [Ca^2+^]cyt in one cell activates Ca^2+^ channels on the membranes of intracellular stores (specifically, TPC1 on the vacuolar membrane), leading to additional Ca^2+^ release from the store and further amplification of the Ca^2+^ signal [129]. This process can propagate from cell to cell, creating a self-sustaining Ca^2+^ wave.

However, TPC1-mediated CICR alone cannot explain the diversity of observed Ca^2+^ waves. This prompted researchers to consider the interaction of Ca^2+^ signaling with other signaling systems, primarily ROS [135].

The specificity of Ca^2+^ signaling is ensured at two levels. The first level is represented by Ca^2+^ signatures—spatiotemporal patterns of [Ca^2+^]_cyt_ changes that encode information about the type and intensity of the stimulus. The second level is provided by Ca^2+^ sensors and their targets: different sensors have different sensitivities to Ca^2+^, different binding kinetics, and different sets of target proteins, which allows Ca^2+^ signatures to be decoded into specific cellular responses.

#### 5.4.3. ROS-Assisted Ca^2+^ Wave

Modern studies have shown that the systemic Ca^2+^ wave is not an isolated signal but is closely linked to the reactive oxygen species wave [56,136]. It has been proposed that systemic signaling in plants is mediated by a ROS-mediated mechanism of Ca^2+^-induced Ca^2+^ release, in which ROS production by the NADPH oxidase RBOHD and Ca^2+^ release dependent on the vacuolar channel TPC1 act in a tightly coupled manner [137]

Systemic signals can propagate through different plant tissues or cell types [137]. As noted, the injury-induced ROS wave propagates through vascular tissue, whereas the Ca^2+^ wave appears to be confined to the cortex and endoderm. However, depending on the type of stress, the propagation pathways may differ. Direct Ca^2+^ translocation through the xylem has been proposed as one component of a long-range Ca^2+^-dependent network of responses. However, in addition to this apoplastic pathway for Ca^2+^ signaling, there is evidence for systemic propagation of elevated cytosolic Ca^2+^ levels via the symplastic pathway (via plasmodesmata) (Figure 6).

Different signals prefer different routes: ROS and ATP act predominantly in the apoplast. Ca^2+^ and some metabolites are transported symplastically via plasmodesmata. Critically, these systems are not isolated, and their interactions determine both the speed and specificity of the systemic response.

**Models of Systemic Signal Propagation: From Molecule to State Models.** Have been proposed to describe the interplay between ROS and Ca^2+^ in systemic signaling [137]. Local stress causes an increase in [Ca^2+^]_cyt_ and activation of RBOHD. RBOHD-produced ROS activate plasma membrane Ca^2+^ channels, leading to additional Ca^2+^ influx. The increase in [Ca^2+^]_cyt_ activates TPC1 on the vacuolar membrane, triggering intra-vacuolar Ca^2+^ release (CICR). The ROS wave and Ca^2+^ wave propagate through the apoplast and cytoplasm, respectively, activating neighboring cells. In neighboring cells, the process is repeated, creating a self-sustaining wave.

ROS-sensitive Ca^2+^ channels in the plasma membrane have been identified electrophysiologically in *Arabidopsis* root cells, and ROS-responsive candidates, such as annexins, have been identified molecularly [138].

**Molecular Modes of ROS and Ca^2+^ Interaction.** The interaction of ROS and Ca^2+^ signaling is mediated by several classes of proteins that serve as “nodes” or “bridges” between these signaling pathways. These proteins primarily include RBOH (NADPH oxidases), which are key integrators of Ca^2+^ and ROS signals. RBOH activity is regulated by Ca^2+^ via the EF-hand domains of RBOH, which bind Ca^2+^ [139], and through phosphorylation by Ca^2+^-dependent protein kinases (CDPK/CPK) and CBL-CIPK complexes [139,140].

Thus, an increase in [Ca^2+^]_cyt_ during stress activates RBOH through both mechanisms, leading to increased ROS production. ROS, in turn, activate Ca^2+^ channels, creating a positive feedback loop.

Another important interaction site is HPCA1 (H_2_O_2_-induced Ca^2+^ increases 1), a plasma leucine-rich repeat kinase that acts as a receptor for extracellular H_2_O_2_. HPCA1 forms two intermolecular disulfide bridges through H_2_O_2_ oxidation, facilitating Ca^2+^ influx and activation of Ca^2+^ signaling. HPCA1 is required for systemic ROS and Ca^2+^ cell-to-cell signaling and plant stress acclimation [141].

**Electrical Signals: The Third Component of the Fast Signaling Network.** Along with Ca^2+^ and ROS waves, electrical signals constitute the third component of the fast systemic signaling network in plants [137]. These three types of signals—Ca^2+^, ROS, and electrical signals—act in concert to rapidly transmit stress information from local tissues to systemic tissues.

The interactions between these signals can be described as follows (Figure 7) [137].

This complex network of interactions allows the three signaling systems to amplify and regulate each other, ensuring effective transmission of the systemic signal to all tissues of the body [39].

#### 5.4.4. Systemic Acquired Acclimation and Ca^2+^ Memory

Systemic acquired adaptation (SAA) is an important mechanism that allows plants to prepare uninjured tissues for upcoming stressors. Systemic signals mediating SAA include Ca^2+^ waves, ROS waves, electrical signals, plant hormones, phosphatidylinositols, and pH signals. Various systemic signals are thought to propagate both upward (e.g., from root to shoot or from lower to upper leaves) and downward [142,143]

The Ca^2+^ wave plays a key role in triggering SAA [27]. Rapid propagation of the Ca^2+^ signal from the site of stress perception to systemic tissues allows the plant to rapidly activate defense mechanisms in organs not directly exposed to stress. Importantly, different abiotic stresses can induce Ca^2+^ waves with different characteristics, potentially providing specificity to the systemic response. Integration of the Ca^2+^ wave with the ROS wave and electrical signals appears to be a prerequisite for effective SAA.

Recent studies have shown that HPCA1 is required for systemic ROS and Ca^2+^ cell-to-cell signaling and plant stress acclimation. This underscores the central role of Ca^2+^ and ROS interactions in systemic adaptation [144].

Ca^2+^ signaling plays a key role in transcriptional reprogramming during abiotic stress. Ca^2+^-induced transcriptional changes trigger molecular mechanisms by which plants adapt and respond to environmental stresses. Ca^2+^ transcriptional regulation mechanisms can directly induce a response through gene and protein activity, or they can be mediated by enhancing Ca^2+^ signals through increased expression of Ca^2+^-responsive genes [94]. Ca^2+^ regulates gene expression in two main ways:

It has been suggested that plants possess so-called Ca^2+^ “memory,” whereby cytosolic Ca^2+^ signatures can be recognized and specifically modified according to previously acquired experience with abiotic stresses [145,146]. A decrease in the cytosolic Ca^2+^ response following repeated exposure to the same abiotic factor has been noted to represent cellular memory, whereby cells are able to retain previously acquired information [147]. This Ca^2+^ memory helps plants maintain balanced Ca^2+^ levels for rapid and effective responses to abiotic stresses. For example, Ca^2+^ memory is one of the strategies for plant adaptation to heat and cold stress [148,149].

## 6. Hormonal Integration of Systemic Plant Stress Responses: From Receptors to the ROS Wave

The plant stress response mechanism is a complex system of interconnected pathways based on the close interaction between various phytohormones and ROS-induced signals [150]. Although the role of the antioxidant system has been well studied, the mechanisms of hormonal control over it remain poorly understood. A complex signaling cascade from stimulus perception to gene expression integrates ROS-dependent pathways and hormonal responses, enabling adaptation to biotic and abiotic stresses by coordinating the activities of hormones such as auxin, gibberellin, cytokinin, abscisic acid, ethylene, brassinosteroids, and salicylic acid [151,152,153]. We decided to focus on these phytohormones in this section. Of course, it must be noted that hormonal balance (the balance of all groups of phytohormones) is important in any process; however, each hormone has its own specific area of action. Moreover, all these hormones exert different effects and can act as antagonists or synergists in response to stress factors, with ROS serving as a critical molecular integrating hormonal signal linking cellular physiological states. Active hormonal regulation of ROS homeostasis mutually modulates the degree of ROS-mediated modifications of upstream signaling components, creating a dynamic bidirectional regulatory circuit [150]. In this context, the synergy between phytohormonal pathways and the antioxidant system is of key importance, where hormones regulate the synthesis and metabolism of ROS [154], and ROS themselves influence the dynamics of hormonal signals under the control of specific transcription factors [155]. Regulation of these processes is ensured through protein phosphorylation, in particular through the activity of mitogen-activated protein kinases (MAPK) and corresponding phosphatases, which direct cellular responses to stress. A special place in this network is occupied by the integration of hormonal signaling with the ROS and calcium ion (Ca^2+^) systems, where NADPH oxidases of the RBOH family act as central nodes linking hormones with the generation of apoplastic ROS and the subsequent activation of cascades of signaling events [156,157].

### 6.1. Abscisic Acid: A Central Regulator of Abiotic Stress

Abscisic acid (ABA) is a phytohormone that plays a key role in regulating plant development and tolerance to abiotic stresses, including drought, salinity, osmotic pressure, and cold [158]. Drought stress increases the production of intracellular ROS, which act as second messengers, triggering adaptive and defensive responses and interacting with ABA and Ca^2+^ signaling pathways. Among the rapid effects of ABA, which occur within minutes of increasing its concentration, is the asymmetric transport of K^+^, Ca^2+^, and anions across the membrane of stomatal guard cells. This slows water entry into the cells, reduces cell turgor, and leads to stomatal closure [158]. At the same time, ABA activates water absorption by roots [159,160].

ABA application exerts a complex regulatory effect on the physiological and biochemical processes of plants under stress, significantly influencing defense mechanisms against oxidative tissue damage [150]. In particular, ABA treatment helps reduce lipid peroxidation by modulating the levels of key metabolites—such as anthocyanins, cysteine, reducing sugars, proline, and polyamines—and by increasing the concentration of soluble proteins, photosynthetic pigments, and ascorbic acid [161]. At the same time, an intensification of antioxidant enzyme activity and a decrease in MDA levels have been observed in various crop species, including rice, wheat, soybean, and sunflower [162].

In addition to biochemical protection, ABA stimulates key indicators of photosynthetic activity: net photosynthetic rate, stomatal conductance, and transpiration rate under drought conditions [163]. Practical examples confirm the effectiveness of this effect: for example, in maize, improved chlorophyll protein synthesis is observed under moisture deficit, and in tolerant hybrids, ABA treatment during flowering results in a significant reduction in MDA levels compared to sensitive varieties [164,165]. At the molecular level, these changes are accompanied by the activation of defense mechanisms, which is confirmed by more intense expression of *LEA* genes in drought-resistant wheat varieties with exogenous application of ABA [166].

#### 6.1.1. Molecular Mechanism of ABA Signaling

ABA signal transduction begins with the formation of a complex between the ABA receptor—proteins of the PYRABACTIN RESISTANCE1/PYR-like/REGULATORY COMPONENT OF ABA RECEPTOR (PYR/PYL/RCAR) family—and protein phosphatases type 2C clade A (PP2CAs) (Figure 8) [100,167,168,169]. This interaction leads to the inactivation of PP2CAs and the activation of SNF1-related protein kinase 2 (SnRK2). Structural studies have revealed a conserved “latch-and-lock” mechanism underlying ABA perception and signal transduction. PYR/PYL receptors are divided into two distinct categories: dimeric receptors (PYR1 and PYL1–PYL3) and monomeric receptors (PYL4–PYL12) [170]. Dimeric receptors have an ABA-binding pocket flanked by a “gate” and a “latch.” In the apo form, the receptors are in an open conformation, allowing ABA access to the binding pocket. Upon binding to ABA, dimeric receptors close the “gate,” creating interaction surfaces that allow PP2C to bind to ABA-bound receptors [167]. This interaction leads to a new conformational change in the protein complex, which blocks the receptor “gate.” Unlike dimeric receptors, monomeric receptors exist in an equilibrium between open and closed “gate” conformations in the absence of ABA and can interact with PP2C in an ABA-independent manner [167].

PP2Cs are key negative regulators of the ABA signaling pathway (Figure 8) [171]. They dephosphorylate kinases such as SnRK2, maintaining their inactive state. When ABA binds to PYR/PYL, the receptors inhibit PP2Cs, allowing SnRK2 to be activated by autophosphorylation. Activated SnRK2 phosphorylates downstream transcription factors, including ABA-responsive element-binding factors (ABF) and ABI2. Phosphorylation of these transcription factors is necessary for their transcriptional activity, which determines transcriptional reprogramming in response to stress factors [172].

In addition to transcription factors, SnRK2 can phosphorylate key components of other signaling cascades, such as RAPTOR (a component of the TOR kinase complex) and ion transporters. Phosphorylation of RAPTOR leads to dissociation of the TOR complex from PYL and the release of ABA signaling under stress conditions. Phosphorylation of ion channels, such as KAT1 (Arabidopsis K^+^ transporter 1), QUAC1 (fast-activating anion channel 1), SLAC1 (slow anion channel), and RBOHD/F (respiratory burst oxidase homologs), determines activities that influence the cellular distribution of the corresponding ions under stress conditions.

Protein kinase FERONIA (FER) has also been shown to be involved in the regulation of ABA signaling by interacting with ABA-responsive protein 2 (ABI2), a member of PP2C that acts upstream in ABA signaling [173,174]. Activated GTPase ROP11 preferentially binds to FER-ROPGEF, which then interacts with ABI2 and activates its phosphatase activity, leading to SnRK2 deactivation and ABA insensitivity (Figure 8) [174]. ABA suppresses ABI2 activity through the receptor pathway, releasing SnRK2 [175,176,177]. However, FER activates ABI2 activity through the ROPGEF–GTPase module, blocking SnRK2 release, leading to suppressed responses to ABA. However, ABI2 interacts with FER and promotes its dephosphorylation and deactivation [173]. Thus, FER and ABI2 form a loop that regulates each other’s activity and participates in ABA-mediated reactions.

#### 6.1.2. Integration of ABA with ROS and Ca^2+^ Signaling

ABA signaling pathways are tightly linked to ROS and Ca^2+^ dynamics, forming a complex regulatory network that enables plants to adapt to abiotic stresses, especially drought [158]. ABA serves as a key phytohormone initiating systemic responses by modulating stomatal conductance and maintaining cellular redox homeostasis. This process is facilitated by the generation and scavenging of ROS. In particular, H_2_O_2_ acts as a second messenger to trigger defense mechanisms by interacting with both ABA-dependent and ABA-independent pathways [178]. Concurrently, Ca^2+^ ions function as critical second messengers during drought stress by regulating ion channels and the activity of protein kinases and phosphatases to mediate rapid physiological adaptation [179]. Notably, ROS-producing enzymes such as NADPH oxidase/RBOH function as a Ca^2+^ channel mediating proton-regulated Ca^2+^ influx, opening a new paradigm for NADPH oxidase research [180]. Recent studies have also identified a plant H_2_O_2_ receptor, HPCA1, which is activated by H_2_O_2_ oxidation, causing an influx of external Ca^2+^ through ROS-sensitive, unknown plasma Ca^2+^ channels, ultimately leading to stomatal closure [106]. Notably, CNGCs are not responsive to H_2_O_2_ [181]. Based on previous studies of RBOH as a proton-gated Ca^2+^ channel in eukaryotes, it is suggested that RBOH may be a specific Ca^2+^ channel for ROS-Ca^2+^ coupling [182]. PP2C, ABI1/ABI2/HAB1, as negative regulators of ABA responses, are directly oxidized by H_2_O_2_ produced by ABA, leading to the inhibition of their catalytic activity and thus to the amplification of the ABA signal in a positive feedback loop [183]. A recent study by Bi et al. [104] (2022) showed that thiol peroxidase PRXIIB acts as a transducer of the H_2_O_2_ signal for ABI2. Specifically, endogenous H_2_O_2_ generated during the activation of the defense system is sensed by PRXIIB via oxidation at the Cys51 residue. PRXIIB then forms disulfide bonds with ABI2, subsequently transducing the H_2_O_2_ signal to ABI2 and inhibiting its phosphatase activity required for stomatal defense in plants [104]. As early as 2006, it was proposed that the Arabidopsis protein glutathione peroxidase 3 (GPX3) functions as a redox transducer through interaction with ABI2 in the ABA signaling pathway and in response to drought [107]. Both PRXIIB and GPX3 are described as interacting with ABI2 and as redox transducers. Also, a study by Li et al. [184] (2024) showed that AhTRXh2 and AhPP2AC2 form a stable complex that promotes the accumulation of ABA and ROS, effectively regulating cell death under conditions of aluminum stress and aging [184].

The interrelationship between these components is supported by their coordinated role in SAA [144]. During thermal or drought stress, ROS waves act in concert with ABA signaling to mediate both immediate and long-term adaptations—the former through rapid oxidative bursts and the latter through hormonal regulation of gene transcription [27]. At the molecular level, ABA perception initiates a “snap-on” mechanism involving PYR/PYL/RCAR receptors, which inhibit PP2C phosphatases and activate SnRK2 kinases. In *Arabidopsis thaliana*, PYR/PYL receptors are classified as dimeric (PYR1, PYL1–3) and monomeric (PYL4–12) [185]. The dimeric forms function as ABA-dependent modulators: in the absence of a ligand, their “gates” remain open, but upon binding to ABA, a conformational change occurs, creating a surface for interaction with PP2C. In contrast, monomeric receptors exist in a dynamic equilibrium between open and closed conformations even without the participation of a hormone, which allows them to suppress PP2C activity regardless of the ABA concentration. Despite the differences in their mechanisms of function, current data indicate that it is the dimeric receptors that play a decisive role in the implementation of a complete biological response to ABA [36,185]. This cascade leads to the activation of anion channels such as OST1, which further regulate aquaporins (e.g., PIP2;1), reducing water permeability in guard cells [184]. Furthermore, ABA influences the activity of antioxidant enzymes through transcription factors such as ABI4, ensuring that ROS levels are maintained within homeostatic limits during stress. By integrating these multifaceted signaling pathways, including pH fluctuations and microRNA regulation, plants can effectively synchronize their metabolic processes to survive under adverse environmental conditions [179].

ABRE family transcription factors also contribute to plant adaptation to water stress. In particular, the AtWRKY75 ortholog (GhWRKY1) plays a key role in regulating drought tolerance in cotton: it represses the expression of ABA-responsive genes such as *RD29A* and *RAB18* by targeting the ABI1 and JAZ1 proteins [186]. Transcriptome analysis confirmed that NCED, ABI1, ABRE, dehydrins, and *LEA* genes are upregulated in chickpea roots under drought stress [187]. Similar mechanisms are observed in millet and maize, where PP2C, SnRK2, ABF transcription factors, and LEA and heat shock family proteins are induced under stress conditions [188]. Importantly, ABA signaling also affects microRNA expression; Thus, in maize, the miR169 family is activated by ABA and *ZmNF-YA* target genes [189]. Thus, ABA regulation is a critical mechanism for drought tolerance in agricultural crops.

ABA-dependent signaling also depends largely on the phosphorylation/dephosphorylation mechanisms of regulatory proteins [190]. Plant genomes contain several thousand kinase genes, the products of which are responsible for the phosphorylation of very specific substrates [191]. At the same time, plant genomes contain genes encoding only a few hundred phosphatases, which may have a broader substrate spectrum than kinases. PP2Cs are the main negative regulators of the ABA signaling pathway, dephosphorylating kinases such as SnRK2 [192].

### 6.2. Auxin: From Development to Stress Response

#### 6.2.1. Auxin Biosynthesis and Physiological Functions

Auxins are a key group of phytohormones that determine the dynamics of plant growth and development at all stages of their life cycle [193]. As “directional hormones,” they are unique in their ability to regulate tropisms, asymmetric cell division during embryogenesis, and organ polarity. In addition to controlling the cell cycle, tissue differentiation (such as vessels and root hairs), and cell elongation through the activation of ion channels, auxins play a critical role in protecting plants from abiotic stress. In particular, exposure to salts or heavy metals activates the RBOH-ROS-auxin systemic signaling cascade: a surge of reactive oxygen species (ROS) triggers the development of lateral roots in unaffected soil compartments, allowing the plant to effectively bypass toxic zones [194].

Drought tolerance is mediated by a complex interaction between the auxin pathway and ABA-responsive genes, which maintains ROS homeostasis and root system architecture in crops such as rice, maize, and *Arabidopsis thaliana* [195,196]. Recent genetic modification research confirms the importance of this mechanism: overexpression of auxin biosynthesis genes (e.g., *YUC6*, *OsIAA6*) or auxin transport regulators (PINs) significantly enhances drought and heat tolerance in various species [197].

Exogenous auxin application regulates drought response in many plants, including wheat seedlings [198]. Moreover, auxin spraying on maize and wheat plants compensates for drought-induced losses [199]. It is reported that indolebutyric acid (IBA) treatment can overcome this root growth suppression in mungbean seedlings [200]. Interestingly, rhizobacteria mitigate drought-induced yield losses by enhancing auxin in plants [201]. Another example of the beneficial effect of exogenously applied auxins is the mitigation of drought-induced spikelet sterility and yield losses in rice [202].

Despite extensive study of the basic mechanisms of indole-3-acetic acid (IAA) metabolism, the complex interplay between auxin signaling, circadian rhythms, and specific microRNAs under extreme stress conditions [203] remains a relevant area for further research.

IAA is a key auxin-type phytohormone, playing a fundamental role in regulating plant growth and development, as well as modulating their responses to adverse environmental factors. Current research highlights the importance of genetic modification of IAA biosynthetic and signaling pathways for enhancing crop tolerance to water stress. In particular, overexpression of *YUC* family genes (e.g., *AtYUC6* in soybean or *YUC7* in Arabidopsis), as well as manipulation of auxin binding factors and PIN transporters, contributes to the maintenance of reactive oxygen species homeostasis and the activation of defense mechanisms [204]. Additional examples include the use of *OsIAA6*, *OsABF1*, and *OsABF57* genes in rice to enhance drought tolerance, and overexpression of auxin-synthesizing enzymes (iiaH, iiaM) or EPSPS to improve water-holding capacity and heat tolerance [205]. Despite significant progress in the study of tryptophan-dependent and -independent pathways of IAA biosynthesis [206], the mechanisms of functioning of these systems under stress conditions remain insufficiently detailed, which determines the prospects for further research in this area.

#### 6.2.2. Phosphorylation in Auxin Signaling

Protein phosphorylation plays a crucial role in all aspects of auxin action, including biosynthesis, transport, signaling, and interactions with other signaling pathways. Auxin transport and phosphorylation of PIN proteins [207]. The AGCVIII PINOID (PID) kinase and its putative functional paralogs WAVY ROOT GROWTH 1 (WAG1) and WAG2 can directly phosphorylate the cytoplasmic domain of PIN proteins. This determines the apical-basal localization and activity of PIN proteins in root cells, while the PP2A phosphatase reduces the phosphorylation level of PIN proteins and alters their localization [208]. Other kinases, in addition to AGC family proteins, are also involved in the regulation of PIN channel phosphorylation. The MAPK kinase module MKK7-MPK6 phosphorylates PIN1 to regulate shoot branching [209]. The calcium-dependent protein kinase CPK29 phosphorylates PIN proteins at specific residues in the hydrophilic loop, which is required for auxin redistribution in a variety of biological processes [210]. The CDPK-related kinase CRK5 can phosphorylate PIN1, PIN2, and PIN3, regulating embryonic development, apical hook development, and root gravitropism, respectively [211].

**Auxin biosynthesis**. ARABIDOPSIS TRYPTOPHANAMINE TRANSFERASE (TAA1), a key enzyme in auxin biosynthesis, is specifically phosphorylated by TRANSMEMBRANE KINASE 4 (TMK4) at Thr101 in response to auxin itself [212]. This phosphorylation inhibits the enzymatic activity of TAA1 and influences plant development. Thus, protein phosphorylation acts as a switch, turning local auxin biosynthesis on and off.

**Auxin signaling via TMK**. Auxin is sensed and converted either by the TRANSPORT INHIBITOR RESPONSE1 (TIR1) pathway or by the TMK pathway [213]. Auxin-dependent signaling via TIR1 is primarily involved in transcriptional regulation, with protein phosphorylation in this pathway being minor. In contrast, TMK (Transmembrane Kinase), along with AUXIN-BINDING PROTEIN 1 (ABP1) and ABP1-like proteins (ABL), trigger a rapid auxin response at the cell surface (Figure 9) [214,215].

TMK1 and TMK4 function redundantly, activating MKK4/5-MPK3/6 to regulate cell division processes during lateral root formation [216]. TMK1 phosphorylates membrane-bound regulator of kinase 2 (MAKR2), a TMK1 inhibitor, leading to feedback regulation by dissociating MAKR2 from the plasma membrane [217]. Furthermore, auxin accumulation can promote TMK1 cleavage and translocation of its C-terminus (TMK1-C) to the cytosol and nucleus. TMK1-C then specifically interacts with and phosphorylates the non-canonical AUX/IAA subfamily of IAA32/34 proteins, stabilizing them to suppress gene transcription. This partially explains the mechanism by which high auxin concentrations elicit specific responses [218]. ARF2, the ARF repressor protein, is also phosphorylated and inactivated by BRASSINOSTEROID-INSENSITIVE 2 (BIN2) kinase and acts as an integrator of BR (brassinosteroids) and auxin in the regulation of gene transcription. Thus, the involvement of protein phosphorylation in this auxin pathway is mainly directed at interactions with other signaling cascades [219].

#### 6.2.3. Auxin Integration with ROS Under Stress

The dynamic interplay between auxin and ROS has only recently been recognized. The canonical auxin signaling pathway includes four main components: the TIR1/AFB receptor complex, the AUX/IAA transcriptional repressors, the ARF factors, and downstream regulatory genes [220]. ROS modulate auxin signaling not only by altering auxin abundance but also by directly modifying signaling components through oxidative post-translational modifications (oxi-PTMs). At the receptor level, ROS and NO promote oxidative modification of conserved cysteines in TIR1 (Cys140/Cys516) and AFB2 (Cys135/Cys511), which facilitates their nuclear import and enhances transcriptional auxin responses. At the repressor level, a rapid ROS burst induces redox-dependent multimerization of IAA3/SHY2 through specific cysteine residues, strengthening its interaction with the co-repressor TPL and enhancing ARF-mediated repression during xerobranching. At the biosynthetic level, H_2_O_2_-mediated sulfenylation of TSB1 at Cys308 inhibits its activity, reducing Trp and auxin biosynthesis and linking redox status to auxin supply. Together, these examples show that oxi-PTMs provide a direct mechanistic interface between ROS and auxin signaling rather than a generic “redox modulation”. Auxin has been shown to induce sequential ROS and NO production in root hair cells, leading to oxidation of cysteine residues in TIR1 (Cys140/Cys516) and AFB2 (Cys135/Cys511) proteins, facilitating their nuclear translocation and triggering auxin signaling [221,222]. Auxin also influences root architecture through denitrosylation of ascorbate peroxidase 1 (APX1), which is responsible for H_2_O_2_ scavenging, with a coordinated counteracting effect between NO production and APX1 modification [223]. Stressful conditions also activate the interaction mechanisms of these molecules: water deficit induces ROS-dependent multimerization of IAA3/SHY2 proteins, which enhances the repression of target genes during lateral root formation [224], while under salt stress, the IAA17.1 protein regulates ROS homeostasis through feedback and flavonol biosynthesis to ensure salt tolerance in poplar [225]. In the context of adaptation to oxidative stress, ROS rearrange auxin gradients by inhibiting the delivery of vesicles with the PIN2 transporter via the ARF-GEF-BEN1 complex, which leads to the accumulation of the hormone in the vascular system and its depletion in epidermal cells [226]. This is supported by data on the effect of H2O2 on plasma membrane stability and the degradation of PIN transporters [227].

Furthermore, auxin transport via ROS-mediated signaling is critical for arsenite defense; aux1 mutants exhibit increased sensitivity due to H_2_O_2_ imbalance [194]. It should be noted that H_2_O_2_ can affect both IAA and ABA levels through tryptophan synthase B subunit 1 (TSB1). Salt-induced sulfoxidation of TSB1 increases ABA accumulation and promotes salt stress tolerance in Arabidopsis [228]. Mutation of the key regulatory factor ITPK4 in *Arabidopsis* leads to increased ROS accumulation and decreased auxin levels in plants under salt stress [229]. In soybean, induced expression of GmSIN1 under salt stress conditions increases cellular levels of ABA and ROS, leading to an amplification of the initial salt stress signal [228].

Ultimately, auxin acts as a central integrator in hormone cross-current networks, interacting with ABA, brassinosteroids, and other regulators. An example of this interaction is the phosphorylation of ABI1/2 proteins by TMK1 in response to high auxin concentrations, which directly stimulates the ABA signaling response.

### 6.3. Brassinosteroids: Growth Hormones as Modulators of Stress Tolerance

Brassinosteroids (BR) play a fundamental role in plant growth, development, and adaptation to stress factors, including water deficit [230]. BRs are polyhydroxysteroid plant hormones that promote active growth and development and improve plant health under stressful conditions. To date, more than 70 BRs have been isolated from plants; brassinolide, 28-homobrassinolide, and 24-epibrassinolide exhibit the greatest biological activity [230]. Scientific evidence confirms that decreased levels of endogenous BR correlate with increased crop sensitivity to drought: for example, tolerant maize varieties exhibit higher levels of typhasterol and 28-norbrassinolide compared to susceptible varieties [231], and studies in tomato highlight the critical importance of BR signaling for plant survival [232]. Mutations in the *C6-OXIDASE* gene in barley lead to BR deficiency, which is accompanied by decreased proline and sucrose content, as well as suppressed photosynthetic activity [233]. Regulation of the stress response is also mediated by transcription factors; for example, the RD26 factor from the NAC family plays a key role in modulating BR signaling to promote drought tolerance [234], and moderate drying can stimulate the biosynthesis of these hormones in young rice panicles.

Exogenous treatment of plants with 24-epibrassinolide (EBR) demonstrates high efficacy in mitigating the negative impact of drought, promoting improved growth parameters and activating ROS scavenging systems. In particular, the use of EBR in pepper in combination with nitric oxide (NO) enhances antioxidant defense, preventing the degradation of physiological parameters and ensuring pollen viability. Furthermore, EBR induces the alternative oxidase pathway [235,236]. Similar beneficial effects have been observed with EBR in maize to compensate for growth and reduce lipid peroxidation [237], and in chickpea, where the combination of EBR with kinetin or GA3 minimizes yield losses [238]. Additionally, it has been found that the use of EBR either alone or in combination with sodium nitroprusside increases the level of antioxidant capacity in tomatoes [239], and the use of BRS analogues such as DI-31 or EBR in combination with cytokines significantly increases the regenerative capacity and overall drought tolerance of crops [240].

The BR signaling pathway has been well studied at all stages, from the receptor to downstream transcription factors. BR perception depends on the plasma membrane-localized leucine-rich repeat receptor kinase (LRR-RK), BR-INSENSITIVE 1 (BRI1), and its homologs BRL1 and BRL3. Without BR, BRI1 remains inactive due to autoinhibition by the inhibitory protein BKI1. BR stimulates heterodimerization between BRI1 and its coreceptor BAK1 (BRI1-ASSOCIATED KINASE 1) and activates the receptor complex via transphosphorylation [241]. Activation of the BRI1-BAK1 complex by BR leads to the activation of downstream cytoplasmic kinases—BR SIGNALING KINASES (BSKs) and CONSTITUTIVE DIFFERENTIAL GROWTH1 (CDG1) [242]. This is followed by activation of the phosphatase BRI1-SUPPRESSOR1 (BSU1) [243]. BSU1 dephosphorylates and inactivates the GSK3-like kinase BIN2 (BR-INSENSITIVE 2), which is a negative regulator of the BR signaling pathway [244]. BIN2 phosphorylates and inactivates the downstream transcription factors BES1 (BRI1-EMS-SUPPRESSOR1) and BZR1 (BRASSINAZOLE-RESISTANT1), suppressing their DNA-binding activity and stimulating their degradation [245]. After BIN2 activity is suppressed and BES1 and BZR1 phosphorylation levels are reduced by PP2A, BES1/BZR1 activate the expression of BRASS-responsive genes to regulate growth, development, and stress response [246]. Thus, the central BRI1-BIN2 module acts as a convergent phosphorylation site for proteins in the BRASSINAZOLE-RESISTANT signaling pathway, either regulating downstream events or receiving signals from other signaling cascades (Figure 10) [247].

#### Brassinosteroids and ROS Association

BRs play a dualistic role in plant physiology: they can mitigate the negative impact of the external environment by activating antioxidant enzymes and reducing ROS concentrations, but can also simultaneously produce ROS to transmit signals necessary for normal development. The mechanistic basis for the dual role of BRs becomes clearer when the two arms of BR-dependent redox regulation are distinguished. The ROS-producing arm relies on the direct transcriptional activation of RBOH1 by the factor BZR1: BZR1 binds to the *RBOH1* promoter and induces the apoplastic generation of H_2_O_2_, a process essential, for instance, for tapetum degradation and pollen development in tomatoes. The ROS-scavenging arm is mediated by the BR-induced activation of antioxidant enzymes (SOD, CAT, APX) and the modulation of the glutathione pool; notably, RBOH1-dependent H_2_O_2_ regulates the accumulation of BZR1 itself via glutathione homeostasis, thereby closing a feedback loop between ROS production and scavenging.

A key node in this loop is the redox-sensing function of BZR1. BZR1 directly senses H_2_O_2_ through the sulfenylation of a conserved Cys63 residue. The oxidation of Cys63 enhances the DNA-binding capacity of BZR1, promotes its nuclear accumulation, and strengthens its interaction with PIF4 and ARF6—ultimately increasing the transcription of BR-dependent genes and promoting cell elongation. Crucially, this modification is reversible: thioredoxin reduces the oxidized Cys63, effectively resetting the threshold for BZR1 redox activation and ensuring the dynamic synchronization of the BR signal with the cellular redox status [248,249]. Thus, BZR1 functions simultaneously as an effector of BR-dependent transcription and as an H_2_O_2_ sensor; this explains why BRs can both generate ROS (via RBOH1) and limit their excessive accumulation (through the induction of antioxidants and a feedback mechanism involving glutathione).

Exogenous administration of 24-epibrassinolide (EBR) further stimulates RBOH activity and ROS production, which triggers feedback mechanisms that increase the level of reduced GSH, ascorbic acid (AsA), and activate MAPK cascades to ensure tolerance to salt, heat, and drought stress in various crops. In parallel, BAK1 participates in ROS generation through the FLS2–BAK1–BIK1–RBOHD cascade; although this cascade was characterized in biotic contexts, BAK1 and BIK1 are also implicated in abiotic stress signaling, and the module illustrates how receptor kinases feed into RBOHD activation [150], and the ZmBSK1-ZmCCaMK module is critical for defense against drought and oxidative stress by enhancing the interaction between components under the influence of BR [105]. Given the specific oxidative capacity of different types of ROS in regulating biochemical processes [250], further studies are needed to thoroughly understand the synergism of ROS family members in governing BR signaling and adaptive responses in plants.

### 6.4. Jasmonic Acid: A Stress Response Regulator and Salicylic Acid Antagonist

#### 6.4.1. Jasmonic Acid Biosynthesis and Signaling Pathway, Physiological Effects

Jasmonates (JA), a class of oxidized lipids (oxylipins) derived from cyclopentanones, act as key signaling molecules regulating plant development and their adaptive responses to biotic and abiotic stress factors [150]. Exposure to extreme temperatures or moisture deficit stimulates the synthesis of jasmonic acid-isoleucine (JA-Ile) in the cytosol; this compound is then transported to the nucleus, where it interacts with the F protein COI1. Formation of the SCF^COI1 complex promotes the binding of COI1 and JAZ proteins, which triggers their degradation via the ubiquitin/26S proteasome system, thereby releasing transcription factors for the activation of the corresponding genes [251]. The bHLH factor MYC2 plays a central role in this signaling pathway, mediating responses to salinity and temperature stress and modulating ROS production via the RBOHD and RBOHF proteins [252]. Activation of MYC2 by JA or its precursor 12-OPDA promotes drought tolerance by enhancing ROS signaling.

Beyond growth regulation, JA, in concert with other phytohormones, plays a critical role in defense mechanisms [205], with genes of the WRKY, MYB, and MYC families determining the effectiveness of stress management [253]). Studies confirm the presence of induced expression of JA biosynthetic genes in drought-tolerant lines of chickpea [254] and soybean [255]. Of particular importance is the crosstalk between the JA and ABA pathways under water stress: the interaction of PYL with the JAZ-MYC2 complex coordinates the balance between defense responses and metabolic reprogramming [256]. The synergy of these hormones is necessary to minimize oxidative damage via the ascorbate-glutathione cycle [257] and ensures the functional convergence of stomatal closure and leaf senescence processes [258]. JA has been shown to induce endogenous ABA in citrus roots for drought tolerance [259], and the PYL5 receptor interacts with the JA-responsive factor MYC2, influencing its regulatory genes [260]. Furthermore, the interaction between JA and cytokinins (CKs) is critical for xylem development; disruption of the JA pathway in coi1-1 and jar1-1 mutants results in defects in additional xylem formation and reduced drought tolerance [261]. In turn, activation of the OsBWMK1 MAPK in rice upon exposure to JA and drought indicates a role of JA in the regulation of physiological responses, including the control of stomatal conductance [262].

#### 6.4.2. SA-JA Antagonism in ROS Wave Regulation of Stress

One of the fundamental mechanisms of hormonal regulation of stress responses in plants is the antagonistic interaction between SA and JA, which plays a crucial role in modulating the ROS wave—a self-sustaining ROS signal. Within this mechanism, JA acts as an inhibitor, suppressing the intensity of the ROS wave upon damage or stress, while SA performs the opposite function, amplifying this signal. This antagonism is critical for maintaining a balance between defense responses to biotic and abiotic environmental factors.

Experimental data in *Arabidopsis thaliana* demonstrate that simultaneous exposure to extreme temperatures suppresses the ROS wave due to the accumulation of both hormones; this effect was abolished in the *aos1* mutant, which is defective in JA biosynthesis, supporting the hypothesis that JA blocks the initiation of this wave. Similar patterns are observed with direct application of phytohormones: JA suppresses, while SA enhances, the ROS wave in response to cold stress or mechanical damage [263]. Specifically, in the coi1 mutant, high basal JA levels effectively block the action of SA, confirming their mutually opposing effects.

The practical significance of this interaction is confirmed by studies in maize [264], where combined application of JA and SA significantly reduces oxidative damage by decreasing MDA levels and lipoxygenase activity while simultaneously increasing proline and soluble sugar concentrations.

Furthermore, the combined action of JA and SA is a key factor in drought tolerance, as demonstrated in tomato through analysis of the s*pr2*, *flacca*, and *nahG* mutants. Studies confirm that levels of JAs, lipid-derived signaling molecules belonging to the oxylipin family [182,265], play a crucial role in plant adaptation to water stress and regulate a wide range of biological processes, including trichome development, stomatal opening, and the cell cycle [266]. Importantly, JA signaling is mediated through mitogen-activated protein kinase (MAPK) and closely interacts with GSH, while SA has a negative effect on this process [267]. For example, methyl jasmonate induces the accumulation of ROS and NO in stomatal guard cells, promoting stomatal closure via the OST1 pathway [268]. This JA-dependent promotion of ROS accumulation illustrates that the role of JA is not uniformly inhibitory. Rather, JA can act as a positive regulator of local RBOH-dependent ROS production in specific contexts—such as guard cells, wound responses, and defense against necrotrophic pathogens—while acting as a negative regulator of the systemic ROS wave that propagates from a local stress site. The distinction is therefore not “JA inhibits ROS” but “JA suppresses the systemic propagation of ROS while potentially promoting local ROS bursts.” This context dependence should be kept in mind when interpreting the SA–JA antagonism discussed below.

Ultimately, the SA-JA interaction represents a complex, context-dependent regulatory hub balancing immediate defense with long-term host viability. This process is further modulated by ethylene (ET), which, via OsEIL3, coordinates transcriptional cascades to mediate specific resistance to biotrophic and necrotrophic infections [269]. Thus, the integration of these hormonal networks allows plants to dynamically adjust immune response priorities depending on the pathogen type and environmental conditions.

#### 6.4.3. Role of NPR1 in SA-JA Antagonism

One of the key proteins underlying the antagonistic interactions between SA and JA is NPR1 (NONEXPRESSOR OF PR GENES 1) (Figure 11) [93]. SA promotes NPR1 monomerization via TRX-h3/5, leading to its nuclear localization and activation of transcriptional responses. JA promotes the S-nitrosylation of NPR1 by S-nitrosoglutathione (GSNO), which retains it as a multimer in the cytosol and prevents the activation of transcriptional responses.

NPR1 is subject to post-translational regulation through ubiquitination, sumoylation, and other modifications. Activation of transcriptional responses by SA suppresses the action of JA and reverses its S-nitrosylation by GSNO reductase. NPR1 is required for systemic ROS accumulation in response to local stress or injury, as well as for local and systemic acclimation. In the absence of NPR1, expression of *PR1* (a marker gene for the SA pathway) is suppressed in local and systemic plant tissues exposed to local stress factors or damage. NPR1 plays a key role as a master regulator of redox reactions in the nucleus and links environmental signals to plant circadian rhythms. Because the ROS wave is accompanied by a redox wave, it can induce diverse transcriptomic responses through NPR1 (and other transcriptional regulators, such as MYB30), which are modulated by the interaction between JA and SA.

### 6.5. Salicylic Acid: An Enhancer of ROS Waves and Systemic Defense

#### 6.5.1. The Role of SA in Stress Responses

Salicylic acid (SA) has traditionally been considered a fundamental regulator of plant defense mechanisms, primarily in the context of systemic acquired resistance (SAR) to biotic threats [89]. However, recent research increasingly points to its critical role in adaptation to abiotic stressors. Moreover, SA is a key regulator of ROS homeostasis, mitigating oxidative damage caused by biotic stress. Upon pathogen attack and SAR induction, the initial oxidative burst, followed by antioxidant accumulation, creates a reducing environment, reducing NPR1 to monomers, which translocate to the nucleus [270].

SA concentrations have been shown to increase in both local and systemic tissues when exposed to temperature extremes (high or low). Specifically, ABA accumulation is essential for the subsequent accumulation of SA and hydrogen peroxide, as well as for the modulation of stomatal conductance when exposed to ozone. JA plays a critical role in local changes, while SA is responsible for systemic responses to this stress factor [95].

Scientific evidence supports the importance of SA in abiotic stress, as it stimulates excess H_2_O_2_ production and increases the activity of antioxidant enzymes, as demonstrated in tomatoes [271]. Since H_2_O_2_ itself induces defense enzymes [272], spraying with it can increase drought tolerance in maize. Furthermore, integration of SA and JA signaling pathways through glutathione modulation has been noted [273]. Studies on maize have shown that pre-treatment with SA before drought stress leads to a decrease in ascorbic acid and glutathione levels due to intensified activity of SOD, CAT, and APX enzymes [274]. Thus, SA triggers a complex signaling cascade that regulates the expression of antioxidant defense genes and ensures general tolerance to the environment. Exogenous application of SA also promotes the accumulation of ABA [275], which is critical for plant survival. The interaction of SA pathways with the ABA, JA, and ethylene metabolic pathways determines the dynamics of growth and stress response [205]. Endogenous SA is required for the detoxification of ROS under drought stress and affects the content of antioxidants such as monoterpenes, chlorophylls, tocopherol, and carotenoids [276].

Genetic studies support these findings: deletion of the SA receptor in tomato increases drought sensitivity [60], and mutant lines with low SA levels exhibit increased susceptibility to water deficit [277]. Transcriptional regulation also plays a key role; For example, the Di19 protein in *Arabidopsis* activates the PR1, PR2, and PR5 genes via CIPK11 to promote drought tolerance [218]. Taken together, these data support the notion that salicylic acid signaling is a crucial component in plant adaptation mechanisms to various environmental challenges.

#### 6.5.2. SA as a Positive Regulator of the ROS Wave

As noted previously, salicylic acid (SA) plays a key role in enhancing the intensity of the ROS wave during stress or tissue injury, acting as a significant positive regulator of systemic signaling. The proposed mechanism for this process is based on the activation of ROS production through RBOH-dependent pathways mediated by the NPR1 protein; this component is required for the propagation of the ROS wave and the initiation of the synthesis of transcripts critical for plant adaptation to stress [278]. Thus, redox signaling intersects with the SA and JA signaling pathways via NPR1, promoting systemic ROS accumulation and coordinating defense responses to abiotic stress or injury. Notably, the biosynthesis of SA itself is also influenced by ROS.

According to current research, H_2_O_2_ functions as a mobile signal for the activation of CCA1 HIKING EXPEDITION (CHE) in systemic tissues, stimulating the synthesis of SA. During pathogen infection, a biphasic redox state dynamic is observed: an initial oxidative phase serves as a signal to trigger plant immunity [279], followed by a reductive phase that directly activates the immune response. Precise control of H_2_O_2_ concentration during these processes is performed by SA through modulation of the activity of the SAP5 and CAT2 enzymes. Pathogen-induced salicylic acid initially inhibits CAT2 activity by direct binding, which helps maintain the oxidative state; however, as SA concentration increases, *SAP5* expression increases, competing with it for binding to CAT2. This protects the functional activity of the enzyme and ensures the transition of the cellular redox balance from the oxidized to the reduced state [280]. Furthermore, RBOH is one of the priority targets of SA in mediating the induction of ROS signals [281]. This fact is supported by studies demonstrating the ability of salicylic acid to stimulate the expression of the *RBOHD* and *RBOHB* genes. This contributes to the explosive release of ROS as part of the ETI response, which ensures potato resistance to the PVY virus through the development of a hypersensitive response (HR) [282].

### 6.6. Integration of Hormonal Signals with ROS and Ca^2+^: A Systemic Adaptation Model

NADPH oxidases of the RBOH family act as hubs linking hormonal signals to ROS production. Activation of RBOH by various hormones leads to the generation of apoplastic ROS, which in turn triggers cascades of signaling events.

Hormonal regulation of RBOH occurs through several mechanisms: direct regulation via Ca^2+^ binding to the EF-hand domains of RBOH, phosphorylation by Ca^2+^-dependent protein kinases (CDPK/CPK) and CBL-CIPK complexes, transcriptional regulation of *RBOH* gene expression by hormonal transcription factors (e.g., BZR1 for RBOH1) (Figure 12). Thus, RBOH serves as a convergence point for Ca^2+^ signaling, hormonal signals, and ROS production, facilitating the integration of various signaling pathways into a single systemic response.

The ROS surge is a self-sustaining process of cell-to-cell ROS production mediated by RBOHD. Hormones play a key role in regulating the initiation, propagation, and amplitude of the ROS surge. ABA acts as a positive regulator of the ROS surge, especially under drought and heat stress conditions. ABA-induced activation of RBOH via SnRK2-dependent phosphorylation promotes ROS generation, which in turn activates Ca^2+^ channels and supports ROS surge propagation. SA is also a positive regulator of the ROS surge, enhancing its intensity through NPR1-dependent mechanisms. JA acts as a negative regulator of the ROS surge, suppressing its initiation and propagation. The hierarchy proposed here is conditional rather than absolute. ABA acts as a temporal initiator of apoplastic ROS bursts primarily under drought and heat stress; SA predominantly enhances the amplitude and propagation of the systemic ROS wave; and JA predominantly suppresses systemic ROS wave propagation. However, JA can also promote local RBOH-dependent ROS accumulation in guard cells, wounding, and defense responses. Thus, the direction of JA action depends on whether one considers local ROS generation or systemic ROS propagation, as well as on stress type, tissue, and timing. The model should therefore be read as a context-dependent modular framework, not as a fixed ranking of hormonal inputs. The antagonistic interaction between SA and JA via NPR1 enables fine-tuning of the ROS signaling depending on the type of stress and physiological context.

BRs can either enhance or suppress ROS signaling depending on the context. BS-induced ROS production via RBOH is important for some developmental processes, but under stress, BSs also activate antioxidant systems, reducing ROS levels (Figure 12).

Systemic acquired adaptation (SAA) is a mechanism that allows plants to prepare uninjured tissues for upcoming stressors. ROS signaling coordinates multiple physiological, molecular, and metabolic responses between different parts of the plant and is essential for SAA.

Hormonal signals play a key role in SAA, acting in coordination with ROS signaling: ABA is essential for SAA under heat stress, acting in coordination with ROS signaling in systemic tissues. SA and JA regulate systemic ROS accumulation and systemic adaptation through antagonistic interactions. NPR1 is a key regulator linking redox signals to hormonal responses in SAA. Thus, SAA represents the integration of hormonal signals, ROS, and Ca^2+^ signaling into a single systemic signaling network, ensuring rapid and efficient preparation of the entire plant for stressful conditions.

## 7. Conclusions and Prospective Research

Modern research shows that hormonal signaling during abiotic stress is a complex integrated network in which individual hormones do not act in isolation but interact through numerous cross-talks. Key elements of this network include protein phosphorylation as a universal regulatory mechanism that ensures rapid and reversible modulation of the activity of signaling components. Different phosphorylation patterns (phosphocodes) determine the specificity of downstream signaling pathways. NADPH oxidases of the RBOH family act as central hubs linking hormonal signals to ROS production. Regulation of RBOH through phosphorylation and Ca^2+^ binding ensures the integration of hormonal and Ca^2+^ signals into a single response.

The SA–JA antagonism should be understood as a context-dependent modulation of ROS wave propagation, not as a universal rule for all ROS pools: JA can promote local ROS accumulation while suppressing systemic propagation, and the net outcome depends on stress type, tissue, and timing. This interaction, mediated by NPR1, allows plants to fine-tune their defense responses depending on the type of stress and physiological context. The integration of hormonal signals with ROS and Ca^2+^ signaling is the basis of systemic acquired adaptation. The coordinated action of these signaling systems ensures rapid preparation of the entire plant for stressful conditions. Despite significant progress in understanding these mechanisms, many open questions remain. How exactly do different hormones coordinate their actions to ensure a specific response to a particular stress? What are the molecular mechanisms that determine whether interactions between hormones are synergistic or antagonistic? How do oxidative post-translational modifications, in addition to phosphorylation, modulate hormonal signaling pathways?

The answers to these questions are not only of fundamental but also of practical importance. Understanding the mechanisms of hormonal integration of stress responses opens new possibilities for improving the stress tolerance of agricultural crops. Genetic engineering of hormonal signaling pathway components, modulation of RBOH activity, and regulation of the SA/JA balance may be effective strategies for creating plants resistant to a range of abiotic stresses under global climate change.

## Figures and Tables

**Figure 1:**
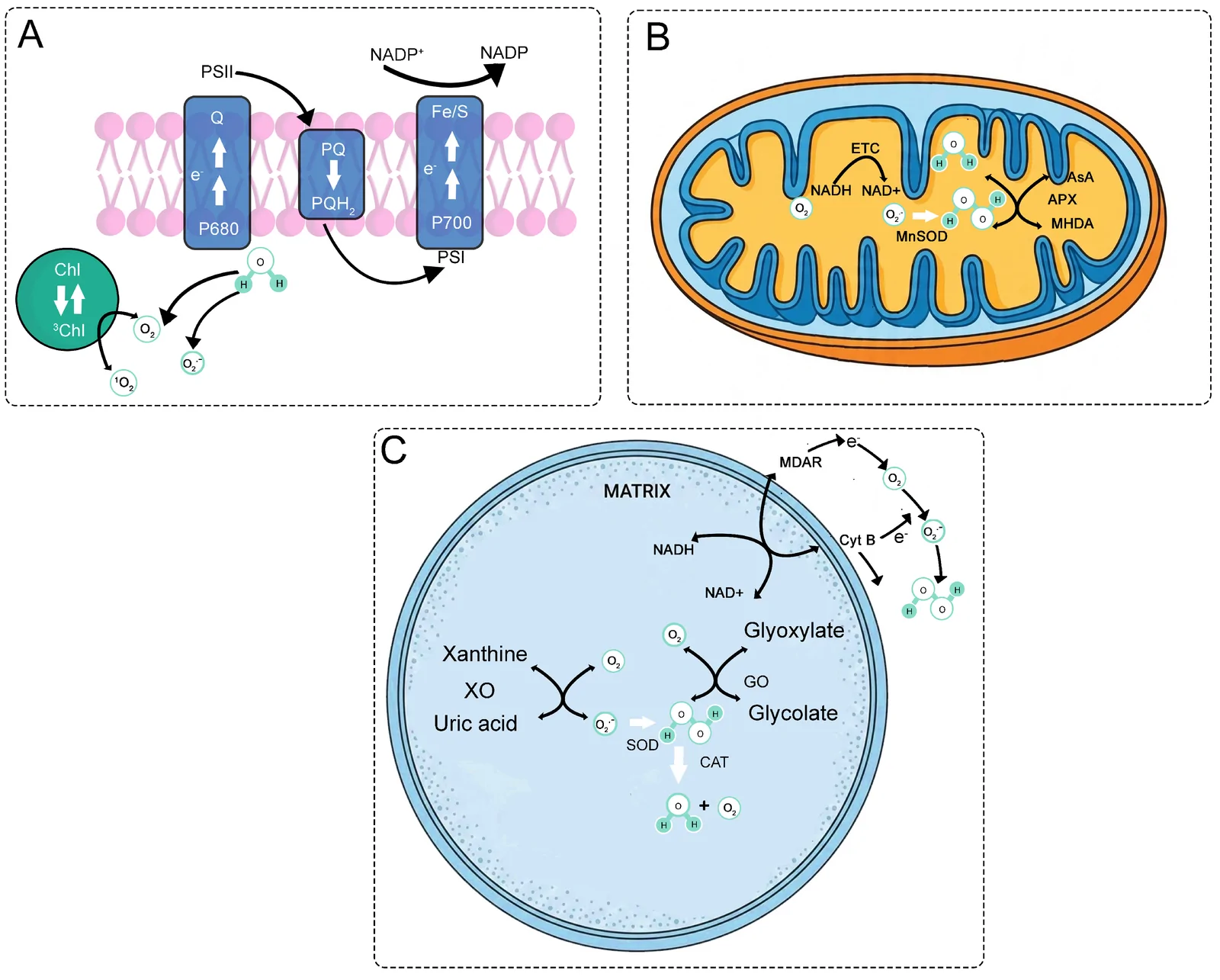
(**A**)—Schematic representation of the processes occurring in the chloroplast during stress, where ROS are generated as a result of CO_2_ inhibition and low water levels are a consequence of stress. PSI-derived O_2_·^−^ is dismutated by SOD to H_2_O_2_, which is subsequently reduced to water by APX in the water–water cycle; this cycle acts as an electron sink under CO_2_ limitation but can also contribute to H_2_O_2_ signaling when its capacity is exceeded. The electron transport chain (ETC) in the photosystem (PS) is the main source of ROS in the chloroplast. Any form of stress leads to a decrease in ROS levels due to ATP synthesis, which leads to a decrease in the ubiquinone (Q) pool. (**B**)—The process of redox reactions in the mitochondria. Several enzymes work together to regulate ROS levels in the mitochondria. (**C**)—Redox processes in peroxisomes.

**Figure 2:**
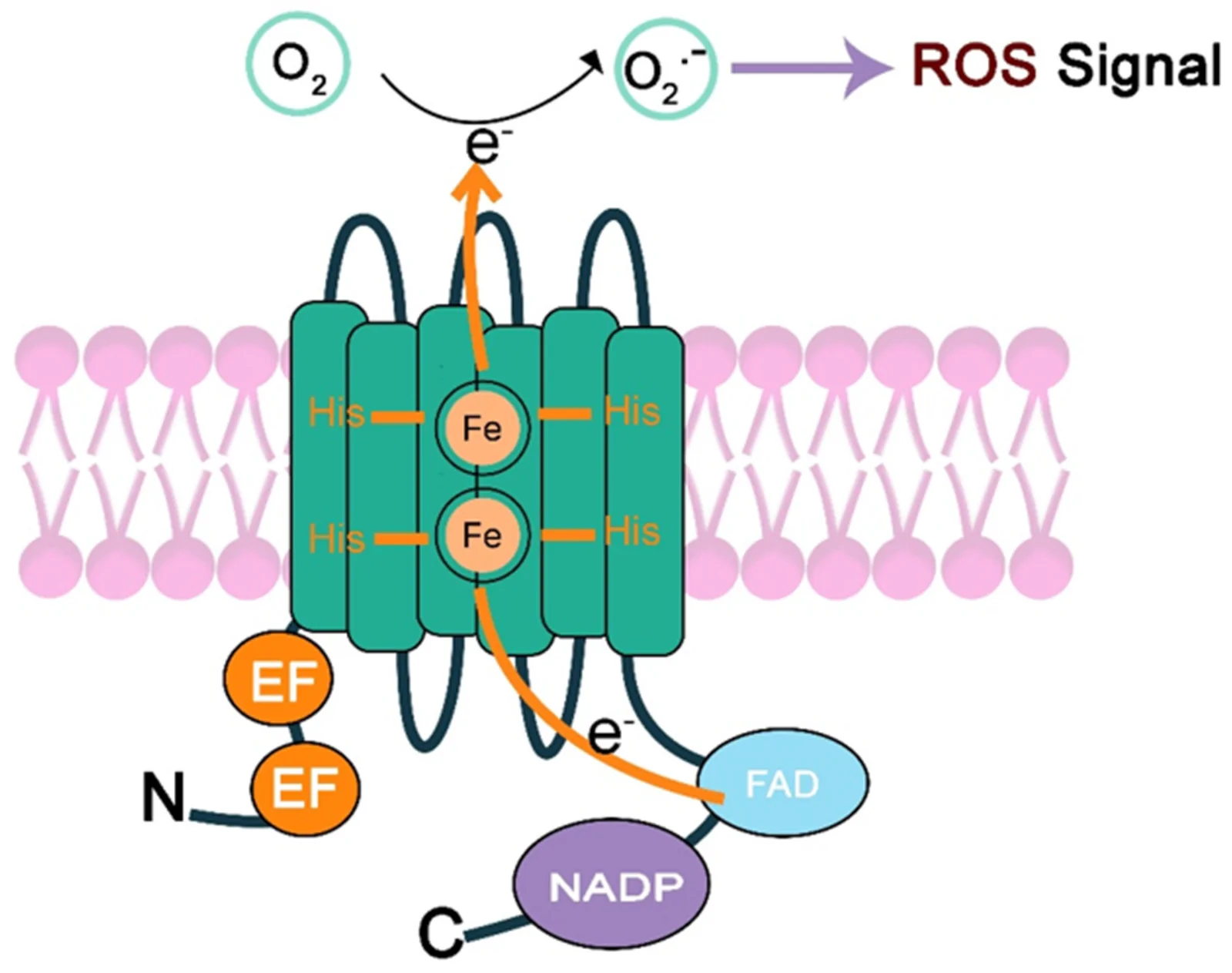
The structure of the transmembrane oxidase RBOH (respiratory burst oxidase homologs) consists of six transmembrane domains. Histidine residues of domains 3 and 5 linked by Fe groups. EF-Ca^2+^-binding sites located at the N-terminus of the protein. Two domains located at the C-terminus of the protein: FAD (flavin adenine dinucleotide) and NADPH (nicotinamide adenine dinucleotide phosphate).

**Figure 3:**
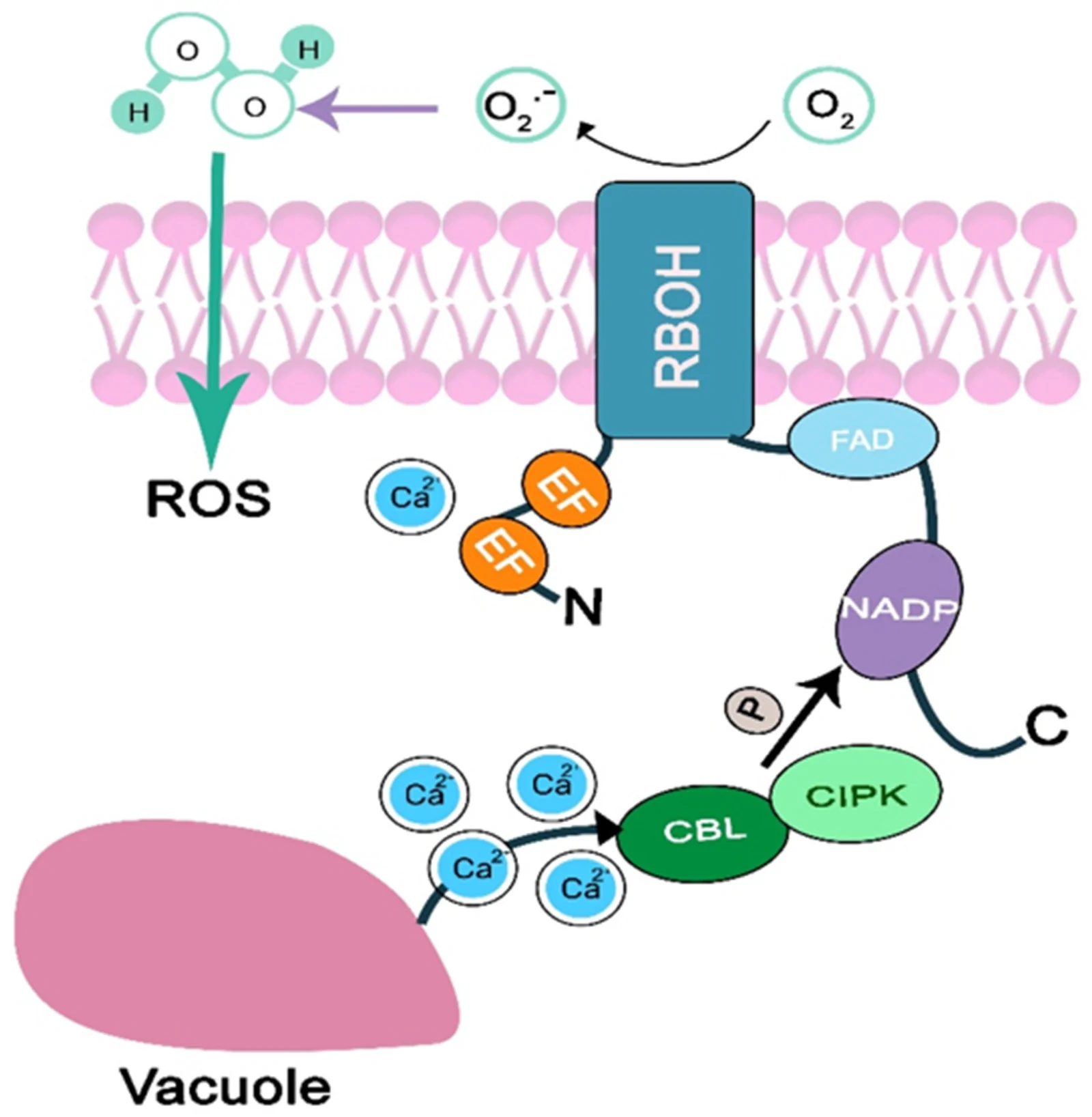
Activation of RBOH oxidase activity through phosphorylation by the CBL/CIPK complex and Ca^2+^ binding by the first EF site.

**Figure 4:**
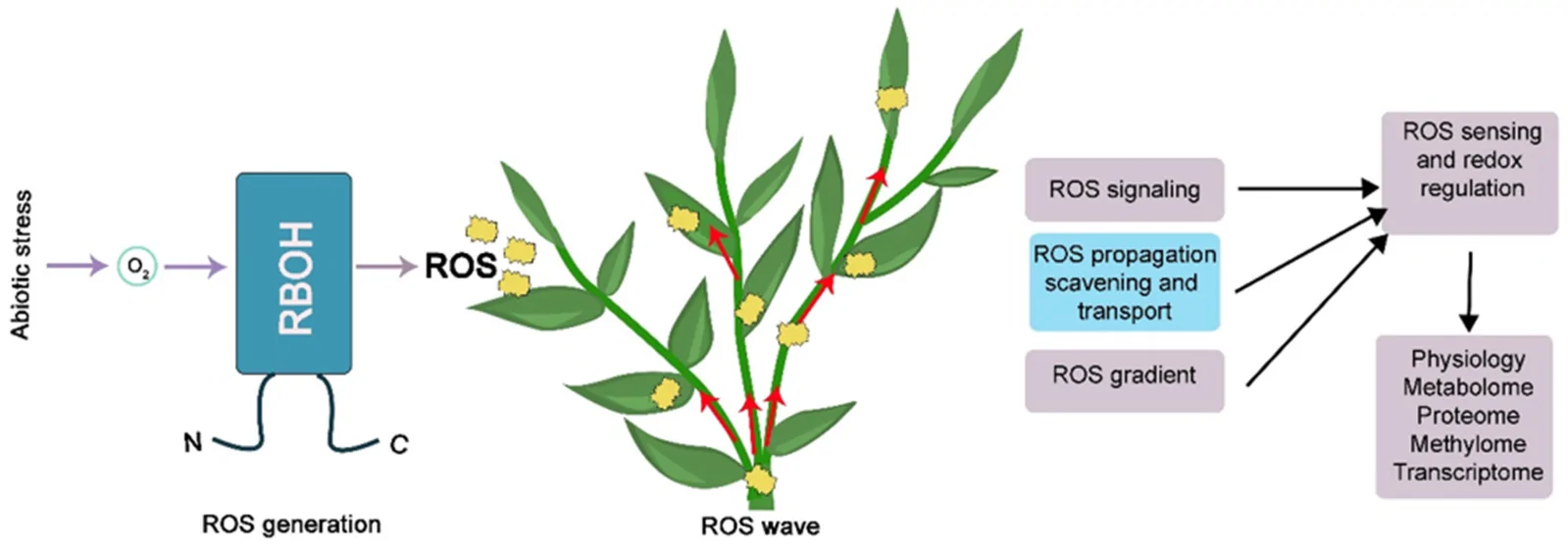
Propagation of the systemic wave of ROS.

**Figure 5:**
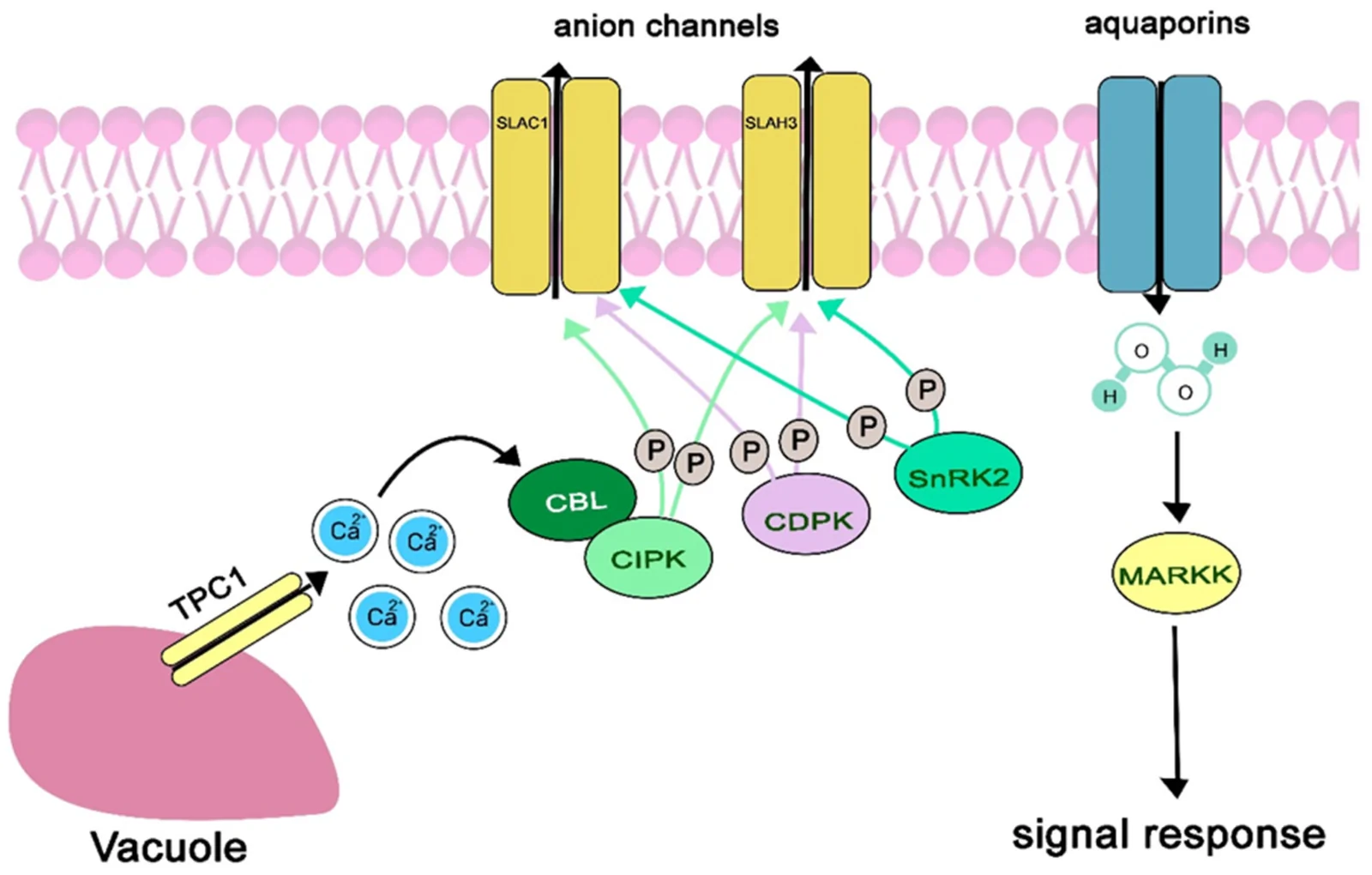
Ion channels and transporters are targets of Ca^2+^-dependent signaling networks. Ca^2+^ signaling mediated by Ca^2+^-dependent protein kinases (CDPK) or calcineurin-like protein kinases (CIPK) on one side, and by nonfermentative sucrose-related kinase (SnRK2) on the other. Activation of SLAC1/SLAH3 anion channels via the kinase leads to plasma membrane depolarization.

**Figure 6:**
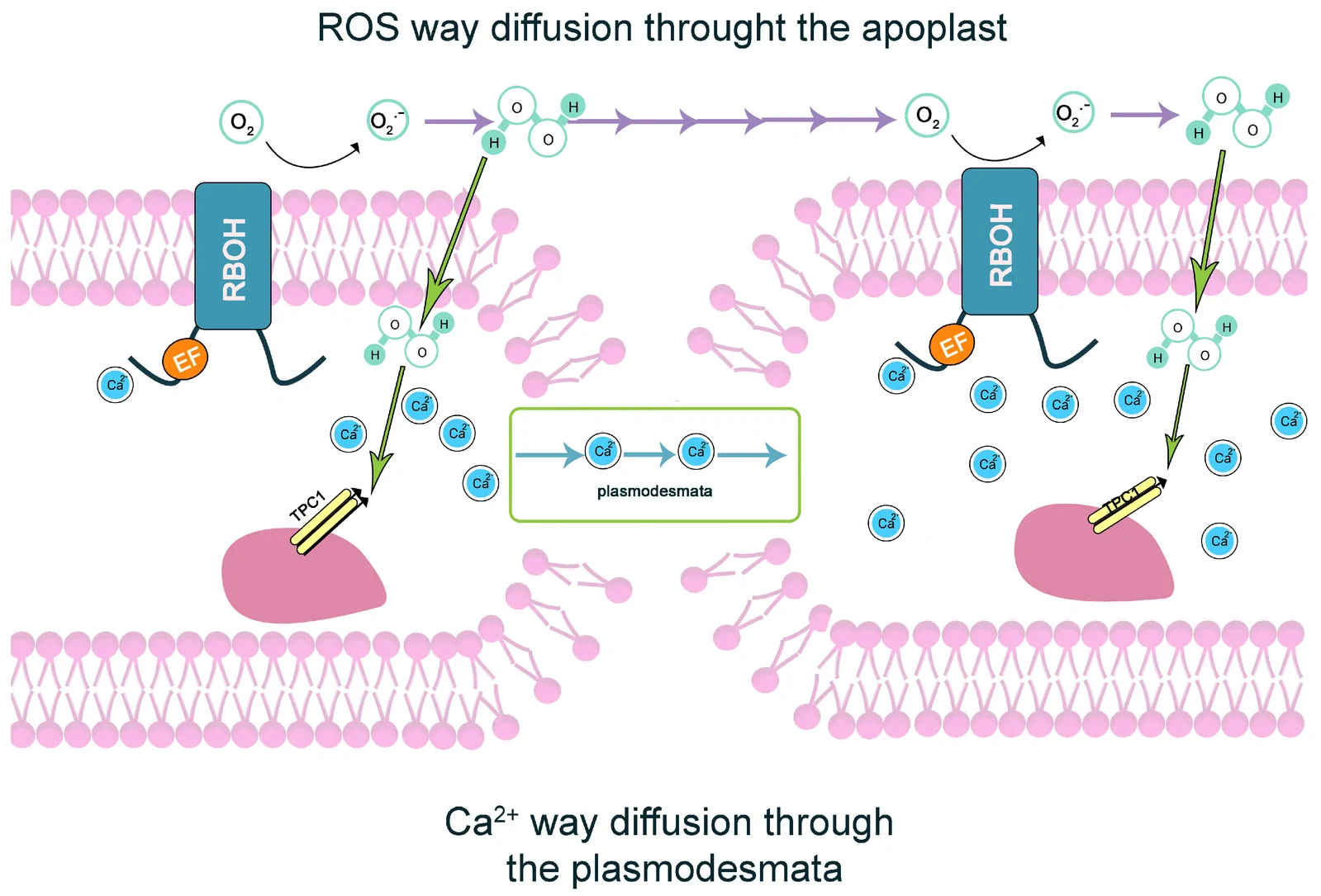
The ROS wave propagates through the apoplast, and the Ca^2+^ wave propagates through the plasmodesmata.

**Figure 7:**
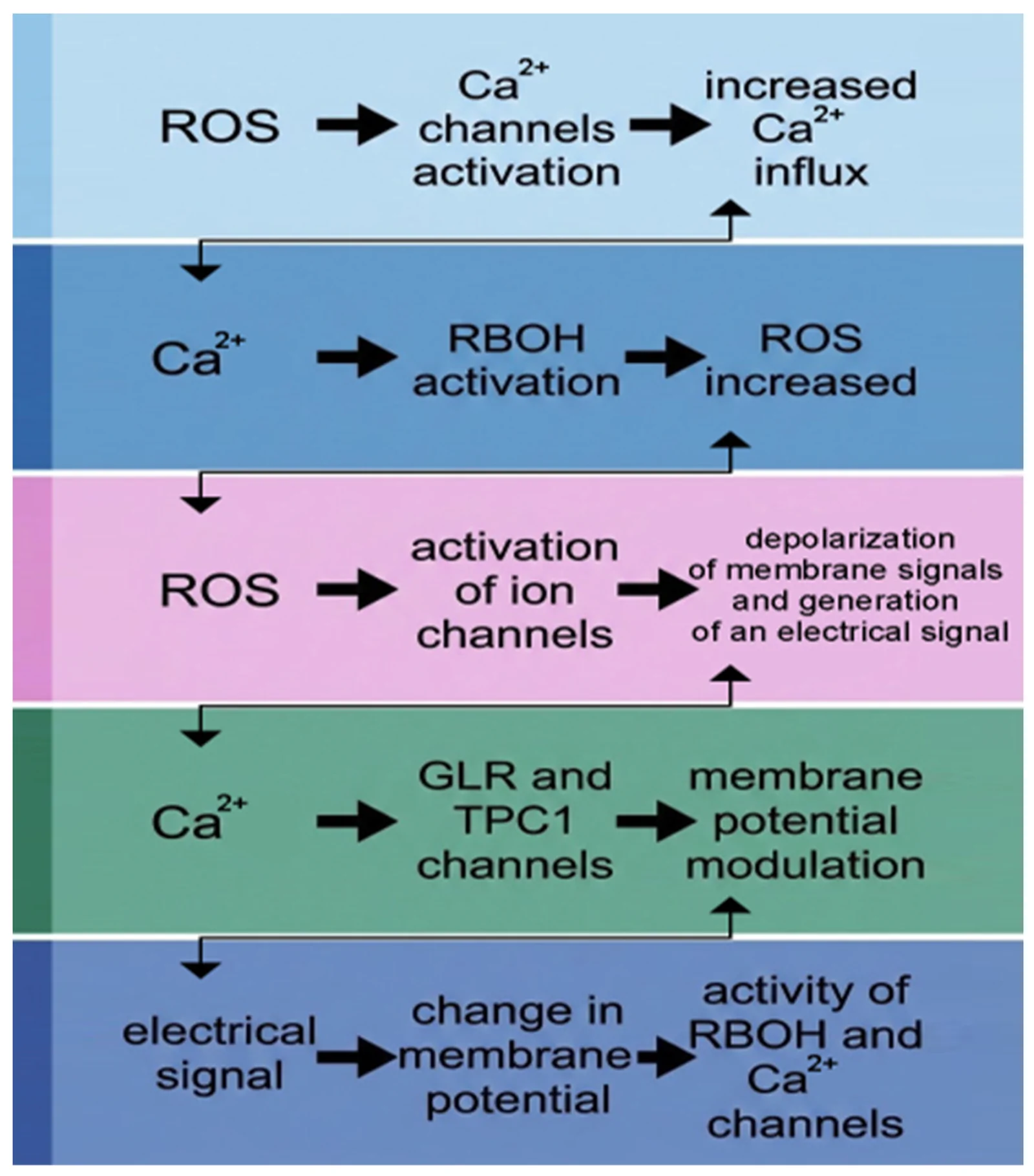
Schematic diagram of the interaction of three waves: ROS, Ca^2+^, and electrical.

**Figure 8:**
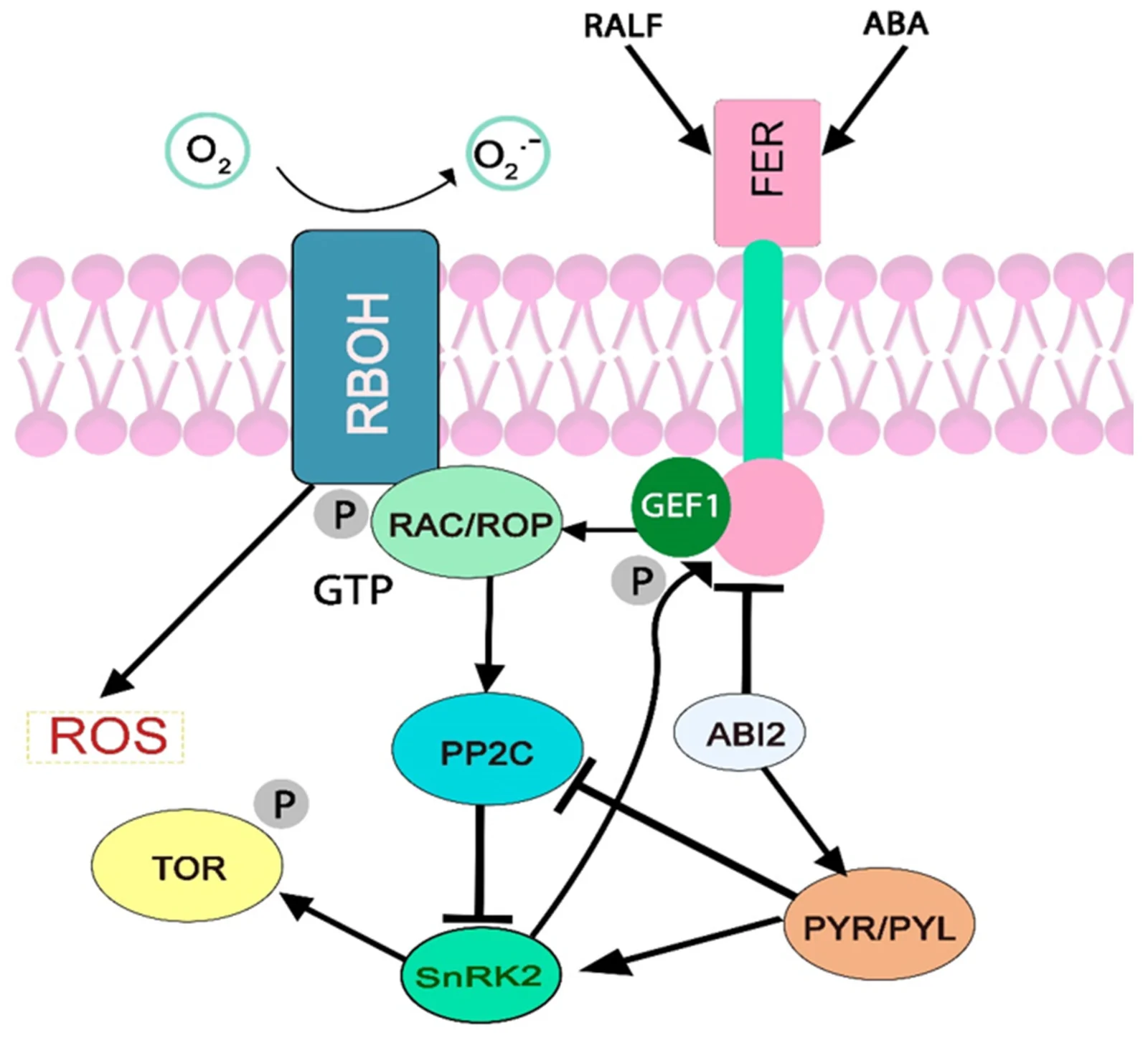
Schematic representation of the role of FERONIA (FER) in the ABA signaling pathway. Sucrose non-fermenting-related kinase 2 (SnRK2) phosphorylates the transmembrane kinase FER, which leads to the activation of an ROS-generating pathway via the GEF1–ROP/RAC signaling cascade. The sucrose non-fermenting-related kinase 2 (SnRK2) phosphorylates the transmembrane kinase FERONIA (FER), which leads to the activation of an ROS-generating pathway via the GEF1–ROP/RAC signaling cascade. The ABA receptor PYR/PYL negatively regulates the phosphatase PP2C and activates SnRK2, which, in turn, activates the kinase TOR (Target of Rapamycin) through phosphorylation.

**Figure 9:**
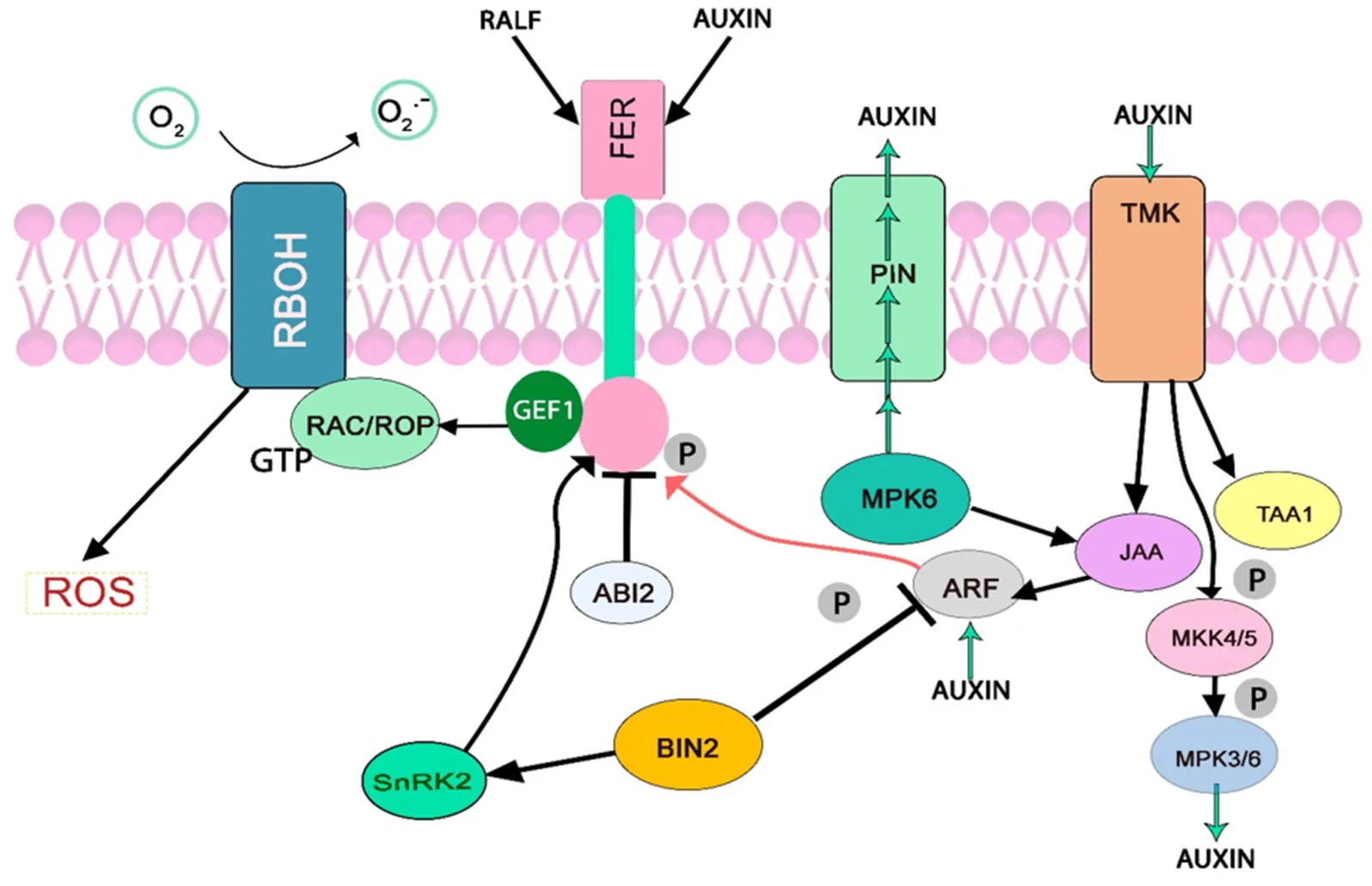
Schematic representation of the role of FERONIA (FER) and TRANSMEMBRANE KINASE (TMK) in the auxin signaling pathway. Auxin signaling is mediated via ARF, a repressor protein that also undergoes phosphorylation and inactivation by the kinase BIN2 (BRASSINOSTEROID-INSENSITIVE 2). The BIN2-SnRK2 pathway leads to the phosphorylation of the kinase domain of FER, which subsequently triggers the activation of RBOH through the GEF1-RAC/ROP module.

**Figure 10:**
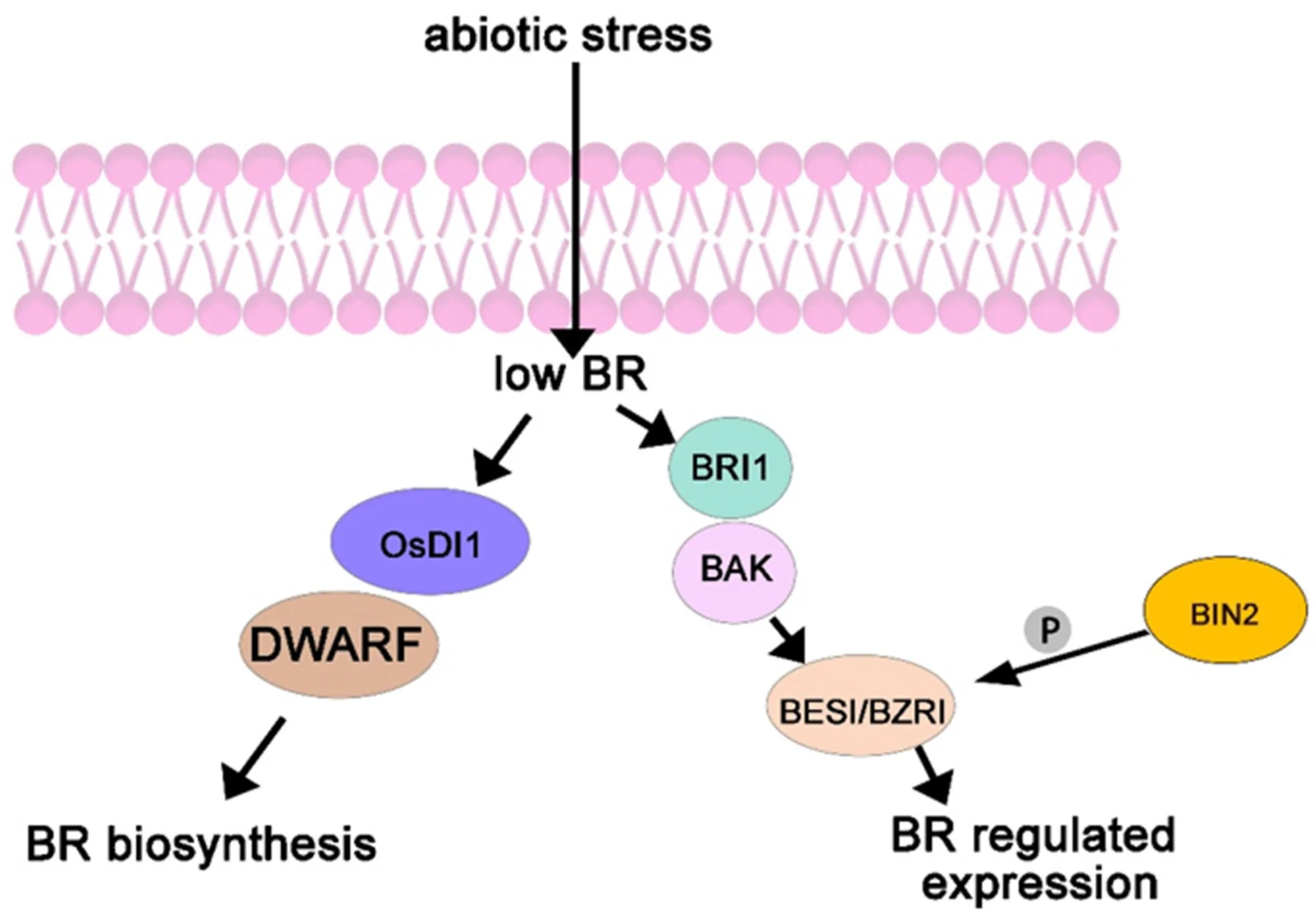
Regulation of BR biosynthesis and regulation of gene expression under abiotic stress through BR kinase complexes. The kinase BIN2 (BRASSINOSTEROID-INSENSITIVE 2) regulates the activation of the transcription factors BZR1 and BES1 through phosphorylation.

**Figure 11:**
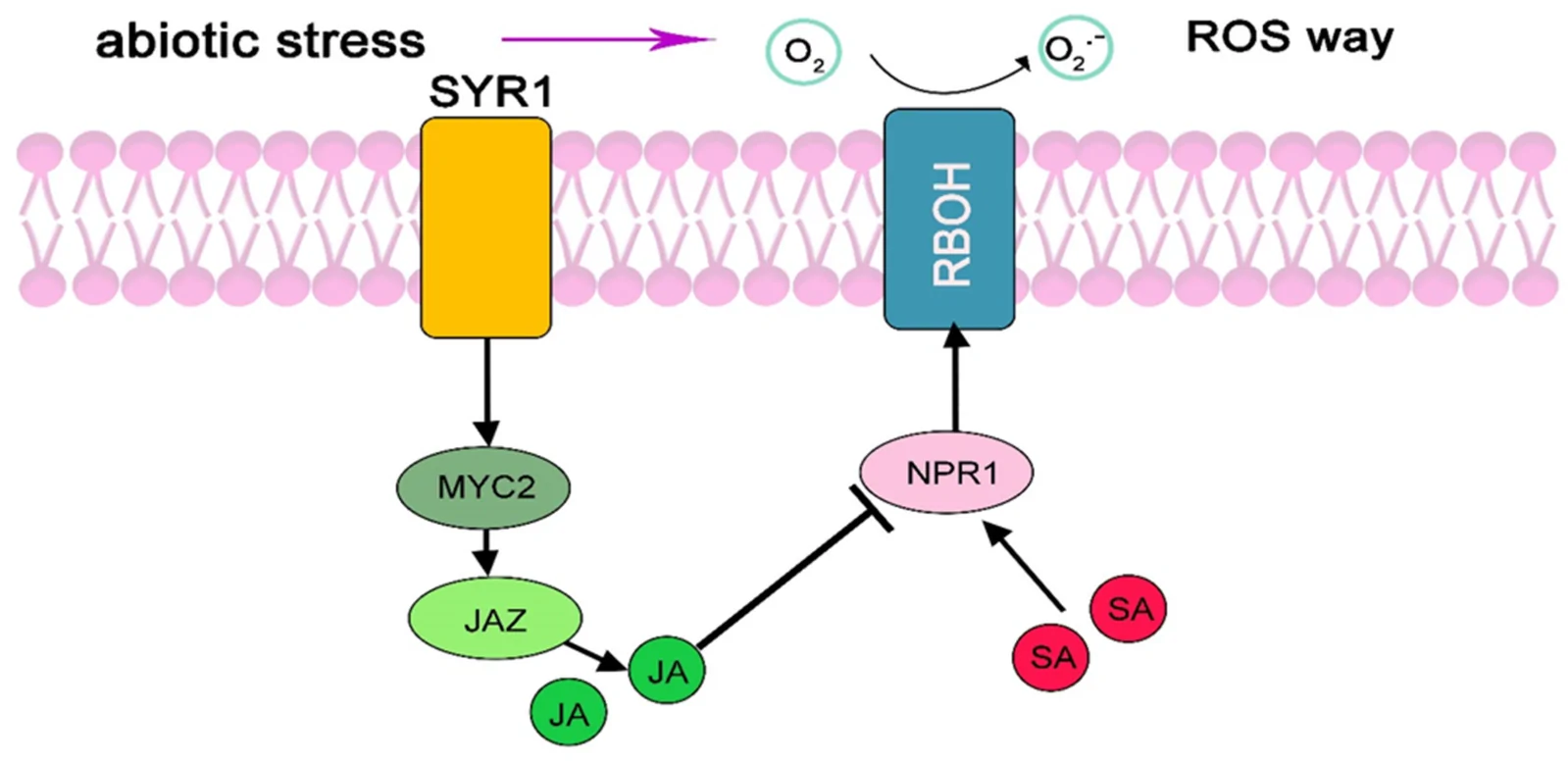
JA signaling pathway and regulation of ROS waves by JA and SA via NPR1. SA activates ROS production, while JA negatively regulates RBOH.

**Figure 12:**
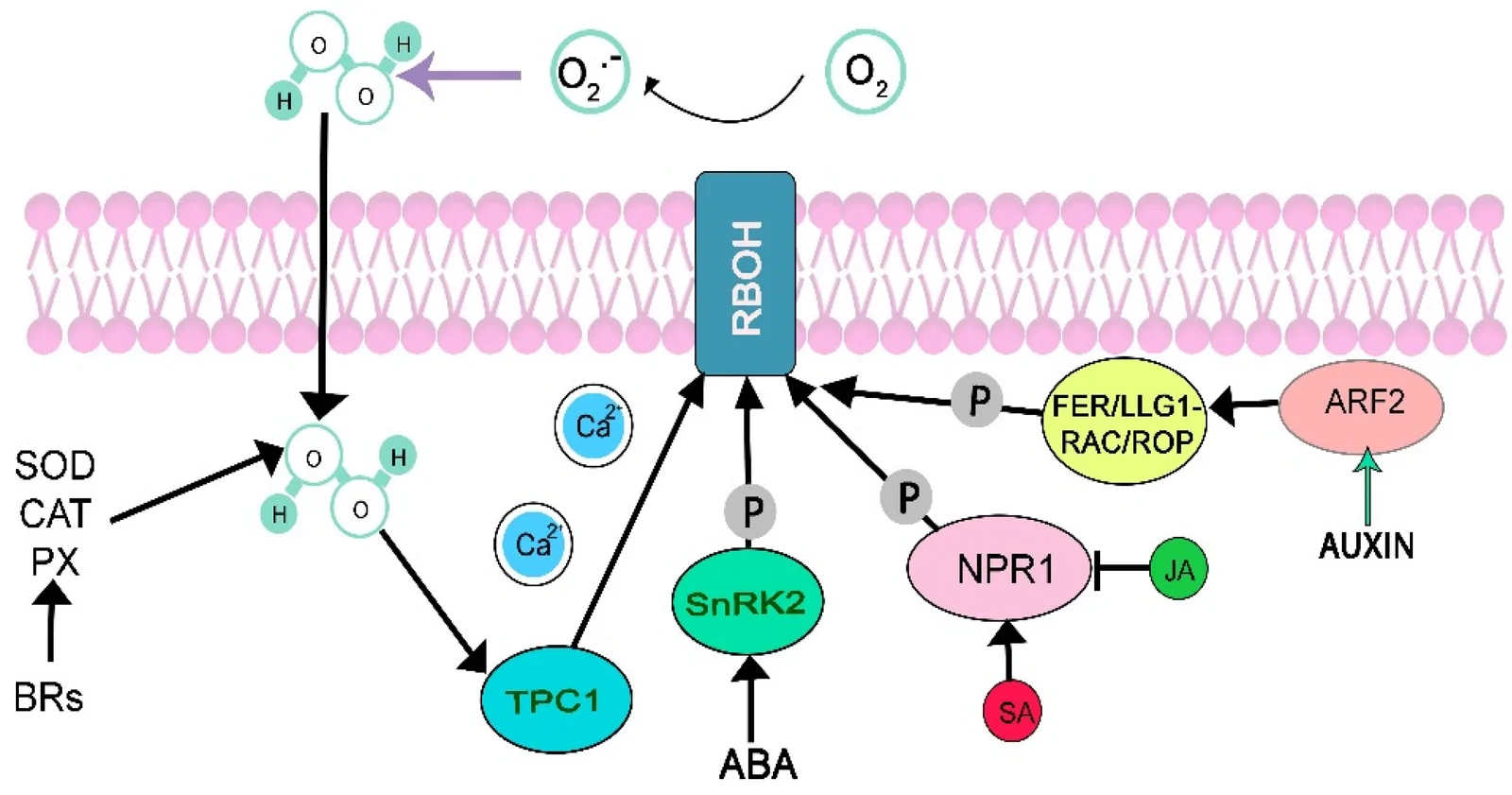
Schematic representation of the main regulators of hormone-mediated ROS signaling.

## Data Availability

No new data were created or analyzed in this study. Data sharing is not applicable to this article

## Abbreviations

The following abbreviations are used in this manuscript:

ABA	abscisic acid
ABI2	ABA-insensitive 2
ABF	ABA-responsive element-binding factors
AOX	alternative oxidase
APX	ascorbate peroxidase
AsA	ascorbic acid
BIN2	brassinosteroid-insensitive 2
BRI1	BR-insensitive 1
BRs	brassinosteroids
BZR1	brassinazole-resistant 1
CaM	calmodulin
CAT	catalase
CBL	calcineurin B-like proteins
COI1	coronatine-insensitive 1
CCaMK	Ca^2+^/CaM-dependent protein kinases
CDPK/CPK	calcium-dependent protein kinases
CIPK	CBL-interacting protein kinases
CML	CaM-like proteins
CNGC	cyclic nucleotide-gated channels
EBR	24-epibrassinolide
ETC	electron transport chain
FER	FERONIA
GLR	glutamate receptor-like channels
GSH	glutathione
HPCA1	hydrogen peroxide-induced Ca^2+^ increases 1
IAA	indole-3-acetic acid
JA	jasmonic acid
JAZ	jasmonate ZIM-domain
MAPK	mitogen-activated protein kinases
MYC2	myelocytomatosis 2
NADPH	nicotinamide adenine dinucleotide phosphate
NPR1	nonexpressor of PR genes 1
OSCA	reduced hyperosmolarity-induced [Ca^2+^]cyt increase channels
PIN	PIN-FORMED proteins
PP2C	protein phosphatases type 2C
PSI	photosystem I
PSII	photosystem II
PYR/PYL/RCAR	pyrabactin resistance 1/PYR-like/regulatory component of ABA receptor
RBOH	respiratory burst oxidase homologs
ROS	reactive oxygen species
SA	salicylic acid
SAA	systemic acquired acclimation/systemic acquired adaptation
SnRK1	SNF1-related protein kinase 1
SnRK2	SNF1-related protein kinase 2
SOD	superoxide dismutase
TIR1	transport inhibitor response 1
TMK	transmembrane kinase
TOR	target of rapamycin
TPC1	two-pore channel 1
